# Multilayer membranes of graphene oxide nanosheets on cellulose filter papers for high-flux water filtration and high proton conductivity

**DOI:** 10.1039/d6ra09284e

**Published:** 2026-10-19

**Authors:** Hon Nhien Le, Ngoc Quang Tran, Nang An Vu, Thi Bich Duyen Luu, Lam Nhu Pham, Thi Bang Tam Dao, Trung Do Nguyen, Chi Nhan Ha Thuc, Hieu Le Van

**Affiliations:** a Faculty of Materials Science and Technology, University of Science Ho Chi Minh City 700000 Vietnam lhnhien@hcmus.edu.vn htcnhan@hcmus.edu.vn lvhieu@hcmus.edu.vn; b Vietnam National University Ho Chi Minh City 700000 Vietnam; c Multifunctional Materials Laboratory, University of Science Ho Chi Minh City 700000 Vietnam; d Center for Innovative Materials and Architectures Ho Chi Minh City 700000 Vietnam; e Center for Materials Innovation and Technology, College of Engineering and Computer Science, VinUniversity Gia Lam District Hanoi 100000 Vietnam

## Abstract

Two-dimensional graphene oxide (GO) nanosheets have emerged as multifunctional units for synthesizing nanostructured membranes for water filtration and proton transport technologies. In this research, advanced GO synthesis were conducted to produce GO dispersions at appropriate concentrations for building primary and secondary layers on cellulose filter papers (Cel). The primary GO layer on the largely porous Cel was formed by vacuum filtration of a GO dispersion 240 ppm. Subsequently, a secondary GO layer was built using a GO dispersion 60 ppm, covering microscale cracks on the primary layer. The multilayer GO membrane on the Cel substrate was compressed and nanostructured for high water permeance (147–240 L m^−2^ h^−1^ bar^−1^), efficient removal of organic dyes (96.7–99.9%), and good performance stability after 15 days in water. Desalination experiments using a Cel/GO/Cel sandwich membrane showed a decrease in NaCl concentration from 2.8% to 0.9% after 12 filtration cycles. Electrochemical impedance spectroscopy measurements were performed to calculate membrane resistances and proton conductivities at 100% RH and various temperatures, leading to the estimation of activation energies. The proton conductivity increased from 1.08–4.82 mS cm^−1^ for Cel/Cel membranes to 17.59–44.83 mS cm^−1^ for Cel/GO/Cel membranes at 25–45 °C. Nanochannels in the GO membranes had a *d*-spacing of 8.5 Å, a water layer, and hydrogen-bond networks for fast proton transport, leading to lower activation energy barrier and higher proton conductivity. The novel multilayer Cel/GO/Cel membrane provides the advantages of low cost, biodegradable substrate, and high performance for water purification and proton conduction.

## Abstract 2

Eco-friendly cellulose papers are upgraded with multilayer graphene oxide membranes to produce nanostructured highways for fast water and proton transports.

## Graphical abstract

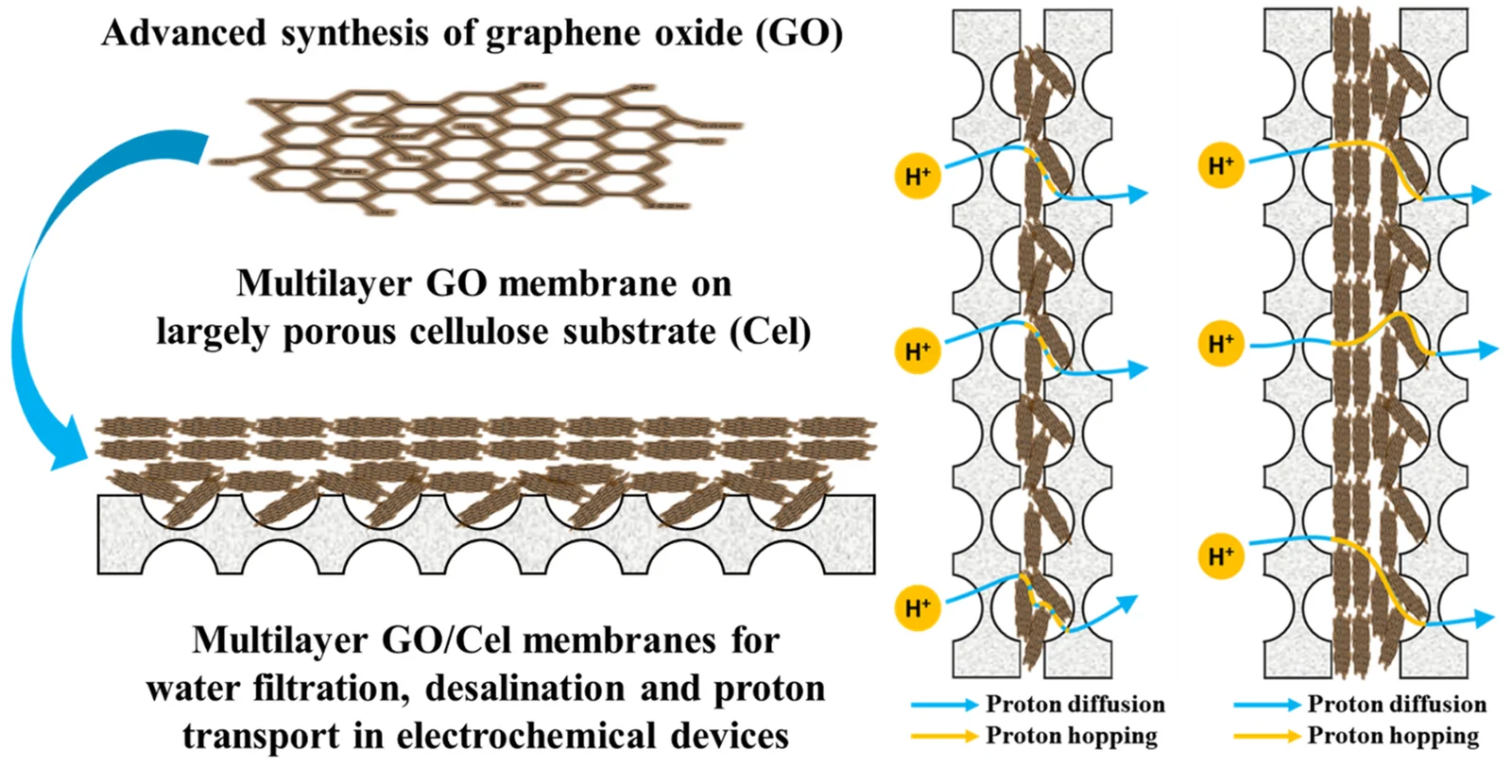

## Introduction

Cellulose filter papers are widely utilized in scientific activities and industrial processes due to their biodegradability, inexpensive cost, and high porosity.^1^ The structure of these papers is a porous network of hydrophilic cellulose fibers, which facilitates efficient filtration and fluid transport.^2^ The cellulose fibers consist of amorphous regions and crystalline cellulose nanocrystals that contribute to mechanical strength of the cellulose fibers, making the filter papers stable for filtration and separation processes. The large pore size (15–20 µm) of regular cellulose filters (Cel) is an advantage for high-flux filtration but also a critical issue for synthesizing thin-film nanofiltration membranes. The relatively large pore sizes limit their ability to retain fine particles and dissolved molecules, resulting in low efficiencies in micro/nano filtrations. Hence, Cel papers are inadequate for fine separations without further modification, such as surface functionalization or nanomaterial incorporation.^3,4^ Recently, thin-film nanocomposite membranes composed of nanofiltration layers on polymer filter substrates have been of great interest.^5^ Although a variety of microfiltration membranes, typically polyester, polyamide, polysulfone, polycarbonate, and polyacrylonitrile, have been used as substrates for fabricating thin-film nanocomposite layers,^6^ the investigation of Cel substrates has not been sufficiently addressed and reported. The large porous and flexible structures of Cel papers are challenges for synthesizing well-structured membranes on the supporting substrate.

Among nanomaterials, graphene oxide (GO) is regarded as a hydrophilic nanoscale building block for the development of next-generation thin-film membranes, particularly in water purification and energy device applications.^7–10^ Over recent decades, the commercial production of GO has progressed substantially, driven by growing demand in various applications, including membranes, coatings, energy storage, and composites.^11^ The synthesis of GO on an industrial scale is primarily based on improved Hummers methods, in which graphite oxidation reaction is conducted using strong oxidants (KMnO_4_ in concentrated H_2_SO_4_), followed by exfoliation to yield GO nanosheets in aqueous dispersions.^12^ Our recent protocol of GO synthesis prioritized safety, efficiency, and scalability through a cascade design system for self-heating reactions, higher chemical efficiency, and better reaction safety for scalable production.^13–15^ The cascade method includes two cascade steps of graphite oxidation reactions. In the first cascade of pouring graphite suspension into Mn(vii)/H_2_SO_4_ solution, exothermic reaction energy is harnessed to heat the graphite/Mn(vii)/H_2_SO_4_ mixture to approximately 50 °C, a safe temperature for effective graphite oxidation. In the second cascade step, the introduction of the graphite/Mn(vii)/H_2_SO_4_ suspension into an appropriate volume of water results in significant exothermic heat generation, facilitating the aqueous oxidation of graphite to graphite oxide (GrO). This step causes the mixture temperature to rise spontaneously from ambient conditions to above 90 °C within a few minutes. By utilizing self-heating exothermic reactions and optimal chemical ratios, the cascade method attains a chemical efficiency of 81.25%, surpassing the chemical efficiency of 62.67% in classical Hummers' method. This represents a notable advancement in synthetic efficiency and energy utilization.^12^

Thin-film membranes composed of GO nanosheets have attracted great interest in the development of membrane technologies owing to their adjustable interlayer spacing, abundant oxygenated functional groups, and hydrophilic properties.^16–18^ In this context, GO membranes exhibit exceptional performance attributed to their two-dimensional layered structure and abundant hydrophilic functional groups, which facilitates molecular sieving, high water flux, and effective rejection of organic contaminants and heavy metal ions.^19–25^ In proton conduction applications, GO membranes are considered potential alternatives to commercial proton exchange membranes, such as Nafion.^26^ The GO structures provide hydrated pathways conducive to both Grotthuss hopping and vehicular transport mechanisms.^27^ Nevertheless, drying-induced cracks and nanomaterial agglomerations present challenges in the design, fabrication, and application of nanostructured membranes.^28^ During the deposition and drying of GO coating on a substrate, solvent evaporation causes significant volume shrinkage and internal stress within the coating. If these drying-induced stresses exceed the mechanical strength of the coating, they lead to the formation of cracks across the coating membrane.

In this manuscript, we report the synthesis and formation of GO membranes on biodegradable cellulose filter paper (Cel). Beside the innovative production of GO building blocks, hydrated graphene-based nanostructures are important for aqueous processability and coating formation.^29–32^ Herein, it is found that the filtration deposition of suitable concentrations of GO dispersion resulted in uniform GO membranes on Cel substrates. The oxidation degree, hydrophilic functional groups, and colloidal aggregates of as-synthesized GO in water are critical for membrane assembly and transport performance. GO membranes were fabricated on a supporting Cel substrate through a controlled layer-by-layer deposition process, resulting in well-ordered laminar architectures with hydrated nanochannels. The obtained GO/Cel membranes demonstrated high water permeance combined with effective rejection of ionic and organic contaminants. In desalination processes, the membranes exhibited considerable salt rejection and structural integrity after many cycles of filtration and washing. Beyond water purification, the proton transport properties of the GO membranes were electrochemically investigated under humidified conditions. The hydrogen-bond network formed by oxygen functional groups and confined water molecules enables efficient proton conduction, yielding proton conductivities comparable to those of proton-conducting membranes in scientific literature. This study demonstrates that as-synthesized GO serves as a multifunctional platform for water nanofiltration, desalination, and proton transport. The insights into structure–property relationships offer guidance for the rational design of next-generation GO-based membrane materials for water and energy-related applications.

## Materials and methods

### Materials

Natural graphite powder supplied by Shanghai Zhanyun Chemical Company was used to synthesize graphite oxide powder. Sulfuric acid (97%, Xilong Scientific), potassium permanganate (99.5%, Duc Giang Chemicals), hydrogen peroxide (30%, Xilong Chemical), and hydrochloric acid (36%, Xilong Chemical) were used as chemical reagents in graphite oxide synthesis. Commercial New Star cellulose filter papers (medium flow grade, 9-cm diameter and pore size 15–20 µm) were obtained from Hangzhou Special Paper Industry Company.

In experiments for water purification, solutions of organic dyes were prepared using methylene blue trihydrate (MB, Xilong Scientific) and rhodamine B (RhB, Sigma-Aldrich), and synthetic seawater was obtained by dissolving sodium chloride (NaCl, Xilong Scientific) in water. Measurements of electrochemical impedance spectroscopy (EIS) employed a nickel foam purchased from Guangdong Canrd New Energy Technology as the electrode material. Pure water obtained from a reverse osmosis system was used throughout the experimental procedures.

### Preparation of graphene oxide

Natural graphite powder was initially used for synthesizing GrO powder and GO nanosheets. The chemical oxidation of graphite was conducted using Hummers reagents (H_2_SO_4_, KMnO_4_, H_2_O, and H_2_O_2_) and the cascade design approach, as detailed in our previous papers.^13–15^ This method specifically employed a moderate quantity of KMnO_4_, maintaining a graphite to KMnO_4_ weight ratio of 1 : 2, and incorporated exothermic cascade steps. The heat generated was effectively used to sustain a self-heating reaction, facilitating the conversion of 15 g of graphite into more than 24 g of GrO powder, with a carbon to oxygen ratio of 1.78.^13^ By optimizing the process design and reagent ratios, the GrO synthetic protocol exhibited better safety, energy saving, chemical effectiveness. Subsequently, GrO powder was dispersed in water and subjected to sonication for 45 minutes to achieve a dispersion of GO nanosheets.

### Fabrication of multilayered graphene oxide membrane on cellulose filter paper

As commercial cellulose filter papers (New Star, medium flow grade, 9 cm diameter) possess a large pore size of 15–20 µm, GO nanosheets serve as 2D building blocks to address the cellulosic porosity. To construct the initial GO layer on the cellulose filter substrate (Cel), a GO suspension with a solid concentration of 240 ppm was prepared by stirring and sonicating 0.1 g of GrO in 325 mL of water for an hour. The resulting 240 mL GO suspension was filtered through a Cel paper using a vacuum pump. At 240 ppm concentration, GO aggregates were captured on the Cel substrate, forming the first nanostructured layer. The vacuum pump and a handheld dryer were used to dry the primary GO layer. While the vacuum pump kept the membrane flat and attached to the funnel, the flow of hot air from the handheld dryer made the membrane dry in a few minutes. With a coating weight of 0.07 g, the Cel paper with the initial GO layer is labeled as GO(0.07)/Cel. Subsequently, 0.1 g of GrO was dispersed and sonicated in 1300 mL of water for an hour, resulting in a 60 ppm GO dispersion. This 60 ppm GO dispersion was vacuum-filtered through a GO(0.07)/Cel membrane to create the second GO layer. The drying of the multilayered membrane was accomplished using the vacuum pump and handheld dryer. With a total GO coating weight of 0.13 g, the multilayered GO/Cel membrane is referred to as GO(0.13)/Cel.

### Direct-flow water filtration for organic dye removal

Cellulose-based membranes of Cel, GO(0.07)/Cel, and GO(0.13)/Cel were prepared for a glass filtration system, including a glass funnel, a vacuum filtration flask, a vacuum pump, and a pressure gauge. A circular membrane was placed on the filter base of the glass funnel with a filter diameter of 4 cm and fixed with an aluminum clamp. Pure water and organic dye solutions, including methylene blue solution 20 ppm (MB20), methylene blue solution 40 ppm (MB40), and rhodamine solution 20 ppm (RhB20), were tested in experiments on direct-flow water filtration. Water permeances at different transmembrane pressures (0.01 bar and 1 bar) were calculated based on the filtrate volumes during a certain filtration time. In the experiment using the low transmembrane pressure of 0.01 bar, the funnel was filled with an aqueous solution to reach the hydraulic height of 102 mm H_2_O, which is approximately equivalent to 0.01 bar. In experiments using a higher transmembrane pressure, a vacuum pump and a pressure gauge were used to create a standard pressure of 1 bar in the direct-flow water filtration system. Filtrates of MB20, MB40 and RhB20 solutions were analyzed with a photospectrometer for ultraviolet-visible light absoprtion spectroscopy (UV-vis).

### Desalination experiments using sandwich membrane structures

GO(0.13)/Cel membranes were selected for direct flow filtration of synthetic seawater. To enhance membrane stability for many cycles of water filtration, a Cel paper was placed on a GO(0.13)/Cel membrane, resulting in a Cel/GO(0.13)/Cel sandwich structure. The sandwich membrane was used in the filtration of saline water containing 2.8% sodium chloride (NaCl). In a typical experiment, 50 mL NaCl solution 2.8% was passed through a Cel/GO(0.13)/Cel membrane at a transmembrane pressure of 1 bar. The sandwich membrane in the funnel was carefully washed with pure water before the next cycle of filtration. The salt concentration in the filtrate was measured using a salinity meter after each filtration cycle. The desalination efficiency for each filtration time was calculated based on the salt concentrations before and after filtration.

### Measurement of proton conductivity of sandwich membranes

Electrochemical impedance spectroscopy (EIS) measurements were conducted using a Gamry Interface 1000 system under the following conditions: potentiostatic mode, DC voltage of 0 V, AC amplitude of 5 mV, frequency range from 1 MHz to 0.1 Hz, and 10 points per decade. Prior to EIS analysis, proton-conducting membranes were pretreated by immersion in 0.1 M H_2_SO_4_ for 1 hour followed by rinsing in pure water for 1 hour, and subsequently stored at 100% relative humidity for 24 hours. Measurements of membrane thickness were carried out using a Mitutoyo electronic thickness gauge. For the measurement, a proton-conducting sandwich membrane was placed between two nickel foam electrodes (0.5 mm thickness, 7 × 7 mm size). The cell was held together by a clamp and put in a plastic box equipped with a temperature controller for EIS measurements at 100% relative humidity (RH) and different temperatures of 25, 30, 35, 40, and 45 °C (see SI Fig. S1). Nyquist plots were fitted using Gamry Echem Analyst software in conjunction with an equivalent fuel cell circuit model for calculating membrane resistance.^33^ Proton conductivity is calculated using the equation:
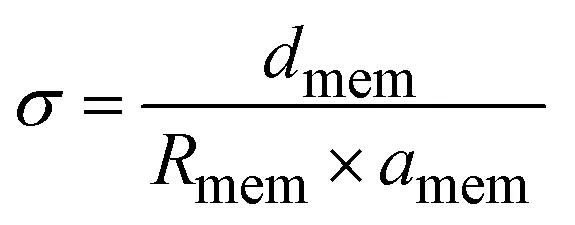
where *σ* is the proton conductivity of the membrane, *d*_mem_ is the membrane thickness, *R*_mem_ is the membrane resistance, and *a*_mem_ is the membrane area.

Activation energy of the proton-conducting membrane was estimated using Arrhenius equation:
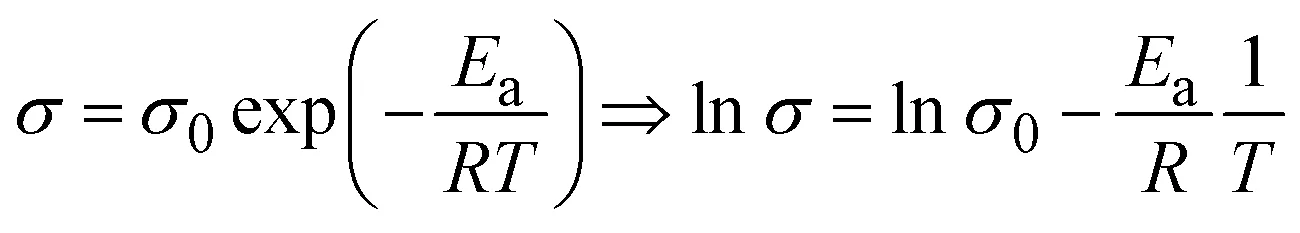
where *σ* is the proton conductivity, *σ*_0_ is the pre-exponential factor, *E*_a_ is the activation energy of the proton-conducting membrane, *T* is the absolute temperature, and *R* is the gas constant (*R* = 8.314462 J mol^−1^ K^−1^). Based on the conductivities at five different temperatures, the plot of ln *σ versus* 1/*T* results in a linear regression function for calculating activation energy.

### Characterization methods

Moisture contents were determined using a moisture analyzer (MX-50, A&D Weighing Company) with a precision of 0.01%. Scanning electron microscopy (SEM) and energy-dispersive X-ray spectroscopy (EDS) elemental analysis were performed using a JEOL JSM-IT200 system. Before performing SEM-EDS characterizations, a thin layer of platinum (Pt) was sputter-coated onto the samples. X-ray diffraction (XRD) patterns were captured with a Bruker D8 Advance diffractometer. Fourier transform infrared spectroscopy (FTIR) and attenuated total reflection FTIR (ATR-FTIR) spectra were acquired using a Nicolet 6700 instrument from Thermo Scientific.

Gas adsorption–desorption analysis of membrane materials was performed using a surface area and pore size analyzer (Anton Paar Nova instrument) equipped with Quantachrome NovaWin software for Brunauer–Emmett–Teller (BET), Barrett–Joyner–Halenda (BJH), and density functional theory (DFT) calculations. The samples were cut and dried at 80 °C before nitrogen gas adsorption measurement at 77 K. The contact angles of water droplets on the membrane surfaces were measured using a Phoenix-MT(*M*) contact angle analyzer. Mechanical characteristics of thin films were assessed with a universal tensile testing machine (YM-H4202-H02, 500 N load cell, Yang Yi Technology Company) in accordance with the ASTM D882-18 standard procedure. Membrane samples of 50 × 6 mm were tested with the tensile speed of 5 mm min^−1^ for determination of tensile strength, elastic modulus, elongation and break energy. A Jasco V-730 spectrophotometer was employed to record ultraviolet-visible (UV-vis) absorption spectra. A pen-type water salinity meter (Total Meter Services Company) was employed to measure the temperatures, pH levels, and NaCl salt concentrations in saline solutions.

## Results and discussion

### Synthesis of graphene oxide dispersions and graphene oxide layers on cellulose substrates

GrO synthesized using the cascade method (Fig. 1a) was ultrasonically exfoliated in water to produce GO dispersions at different concentrations, specifically the higher concentration of 240 ppm (Fig. 1b) and the lower concentration of 60 ppm (Fig. 1c). The multilayer structure of GrO particles was visualized in SEM images in Fig. 1d and e. Owing to the presence of hydrophilic oxygen-containing functional groups, the GrO structures adsorbed water to form water layers in the intersheet galleries. With a water content of approximately 22%, the water molecules in the GrO galleries absorbed ultrasonic waves for vibrations and cavitations, resulting in the exfoliation of GrO into GO nanosheets. The GO nanosheets in the dilute dispersion 60 ppm were better separated into individual nanosheets, exhibiting single-layer nanosheets in TEM images (Fig. 1h and i). In the GO dispersion with a higher concentration of 240 ppm, as the distances between GO nanosheets decreased, intersheet interactions, such as van der Waals attraction, π–π stacking, and hydrogen bonding, became more pronounced, promoting the formation of hydrated GO aggregates (SEM images in Fig. 1f and g). The GO aggregates in the aqueous dispersion 240 ppm were suitable for making the primary GO layer on cellulose filter papers, and the GO dispersion 60 ppm was used for making the secondary GO layer on the primary layer.

**Fig. 1:**
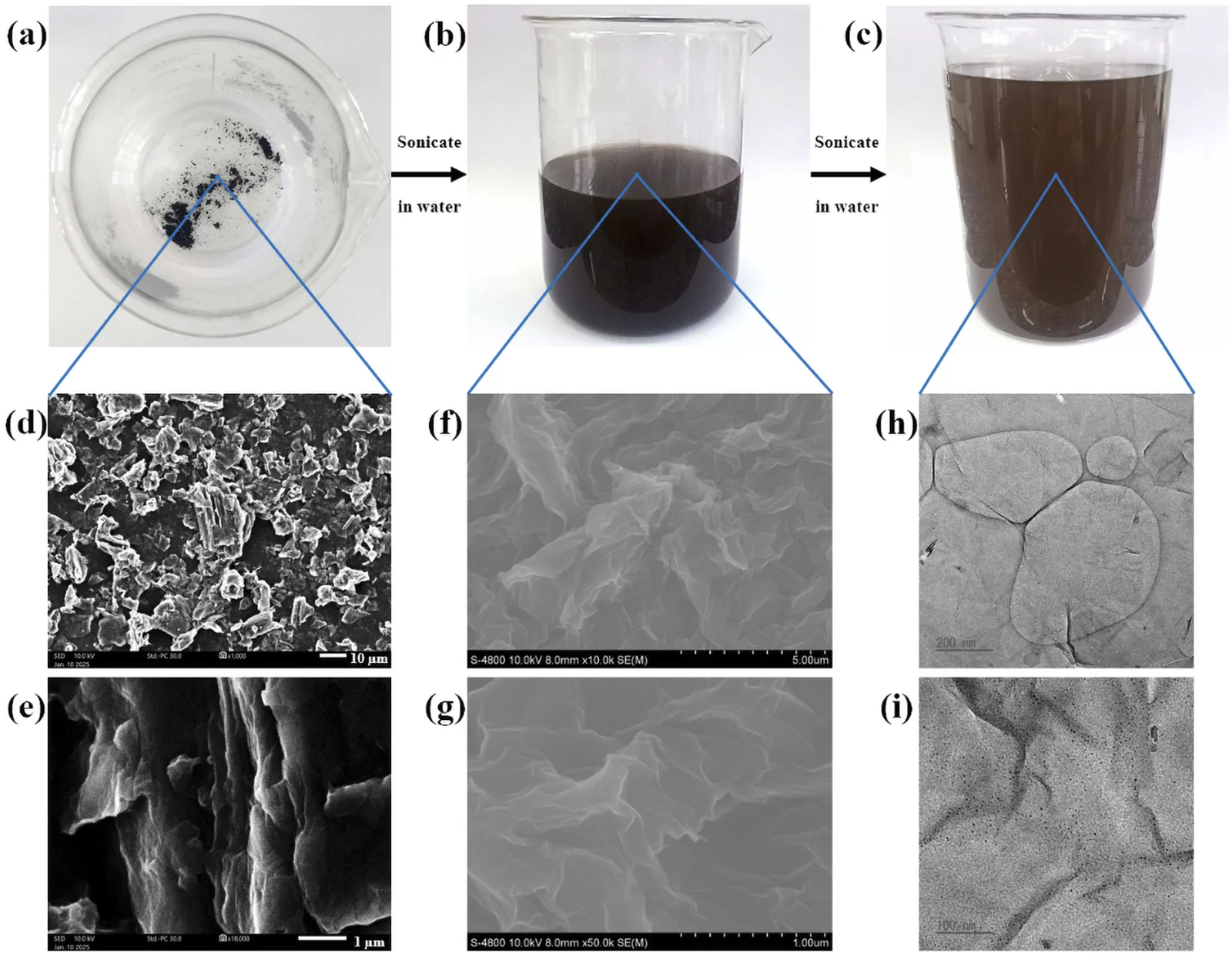
GrO powder synthesized *via* the cascade-design graphite-oxidation process (a) and SEM images of GrO with scale bars of 10 µm (b) and 1 µm (c). (d) 300 mL GO dispersion 240 ppm and FE-SEM images of the GO nanosheets with scale bars of 5 µm (e) and 1 µm (f). (g) 1000 mL GO dispersion 60 ppm and TEM images of the GO nanosheets with scale bars of 200 nm (h) and 100 nm (i).

The microstructures of the cellulose filter paper (Fig. 2a) were characterized using SEM images (Fig. 2b and c), showing the interconnected network of cellulose fibers with a pore size of 15 - 20 µm (Fig. 2b). The SEM image in Fig. 2c shows the surface of a cellulose fiber with a diameter of >20 µm. To fill the largely porous Cel paper, GO aggregates with larger sizes in GO dispersion 240 ppm were retained as the primary layer on a cellulose filter substrate, resulting in a GO(0.07)/Cel membrane (Fig. 2d). Although the primary GO structure played an important role in pore filling and adhesion on Cel substrate (as described in SEM images in Fig. 2e and f), significant fractures in microscale were observed on the surface of the fundamental GO membrane (Fig. 2e). These microscale cracks may facilitate the rate of water flow through the membrane but reduce the efficiency of removing nanoscale and molecular contaminants. Therefore, smaller GO nanostructures in the dilute GO dispersion 60 ppm were introduced to fill the crack spaces, enhancing the nanostructured membrane. SEM images in Fig. 2h and i presented that GO nanosheets of the secondary GO layer covered microcracks on the primary GO layer, resulting in the uniform surface of the multilayer membrane (GO(0.13)/Cel). The prolonged vacuum filtration and vacuum-assisted drying processes also made the GO(0.13) membrane compressed, crosslinked, and adhered to the Cel substrate.^34–36^

**Fig. 2:**
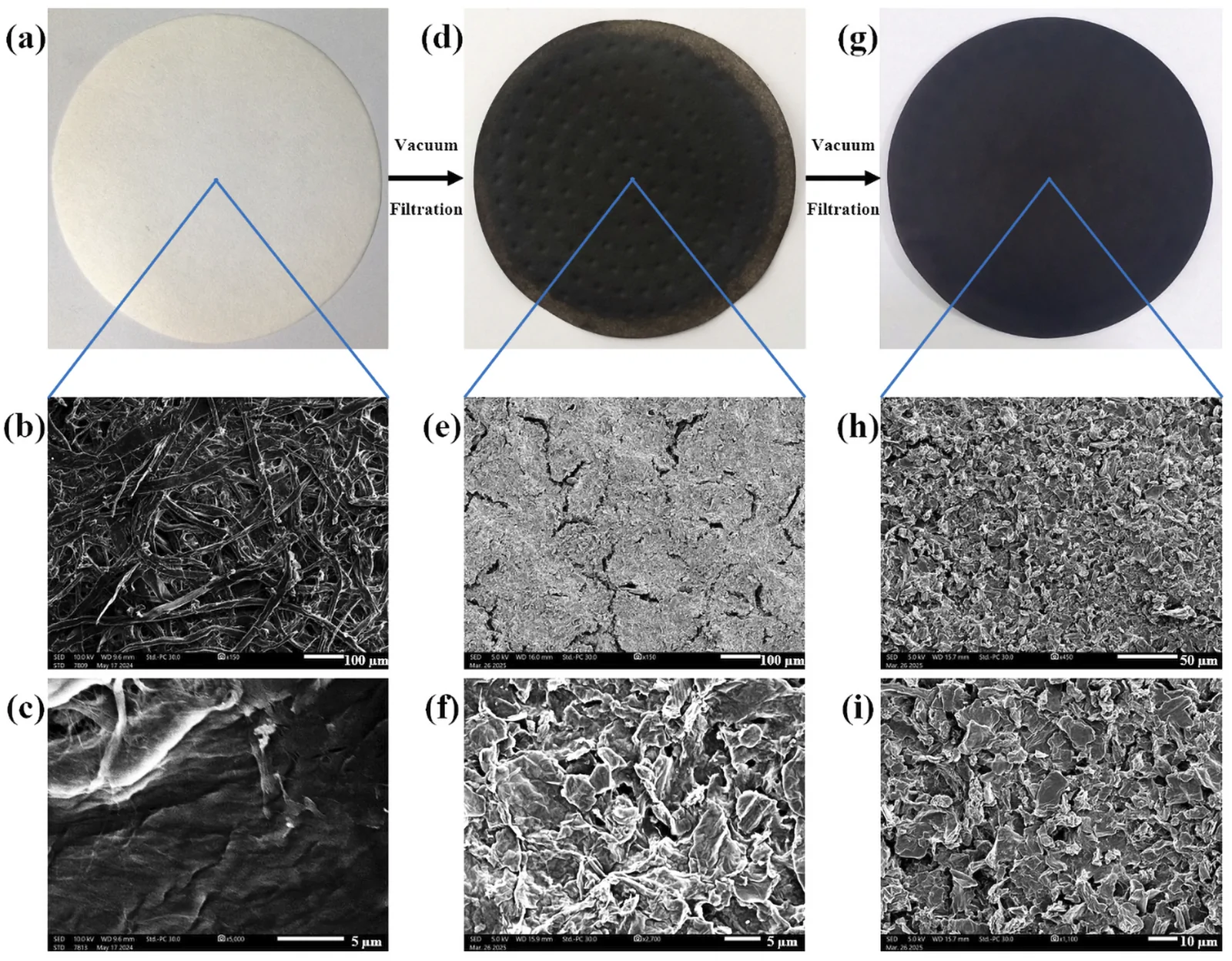
(a) Initial cellulose filter paper (Cel) and its top-view SEM images with scale bars of 100 µm (b) and 5 µm (c). (d) First GO layer on a Cel substrate (GO(0.07)/Cel) and its top-view SEM images with scale bars of 100 µm (e) and 5 µm (f). (g) Multilayered GO membrane on a Cel substrate (GO(0.13)/Cel) and its top-view SEM images with scale bars of 50 µm (h) and 10 µm (i).

Elemental mapping and composition of the GO(0.13)/Cel were analyzed using SEM-EDS characterization (Fig. 3). The SEM image in Fig. 3a shows thin GO nanosheets on the membrane surface, and the integrated SEM-EDS image in Fig. 3b reveals the elemental mapping of carbon (yellow dots, Fig. 3c) and oxygen (red dots, Fig. 3d) atoms. The oxygen atoms were uniformly distributed on the surface, indicating the presence of oxygen-containing functional groups on the GO membrane. According to Table 1, the proportions of carbon and oxygen atoms in the GO(0.13)/Cel membrane were 68.16% and 31.84%, respectively. The obtained *C*/*O* ratio of 2.14, which is slightly higher than those of GO nanosheets (*C*/*O* of 1.78), suggests that the multilayer GO membrane was partially reduced during sonication, filtration, and drying. The assembly of GO nanosheets containing numerous oxygen-containing functional groups in the multilayer membrane formed hydrophilic nanochannels for fast water transport through the membrane.

**Fig. 3:**
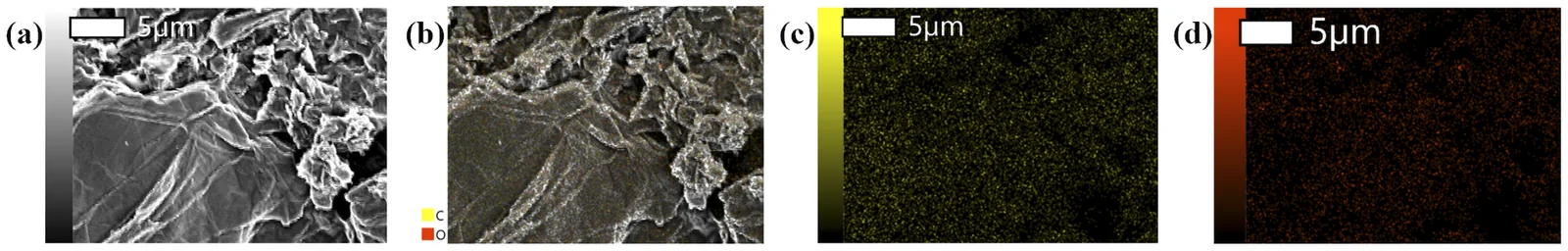
SEM-EDS characterization of the surface of the GO(0.13)/Cel membrane. (a) Top-view SEM image of the surface area characterized by EDS. (b) Combined elemental mapping of carbon (yellow dots) and oxygen (red dots) on the surface area. (c) Elemental mapping of carbon (yellow dots). (d) Elemental mapping of oxygen atoms (red dots).

**Table 1:** EDS results of elemental compositions of initial Cel paper and as-synthesized GO(0.13)/Cel membrane

Materials	C		O	
	(Weight %)	(Atom %)	(Weight %)	(Atom %)
Cel substrate	45.81 ± 1.18	52.96 ± 1.36	54.19 ± 2.47	47.04 ± 2.15
GO(0.13)/Cel	61.64 ± 0.44	68.16 ± 0.48	38.36 ± 0.68	31.84 ± 0.56

### X-ray diffraction, attenuated total reflection infrared spectroscopy, gas adsorption analysis, and contact angle measurement

In Fig. 4a, the XRD pattern of the Cel filter paper exhibited characteristic diffraction peaks at 2*θ* values of 14.9°, 16.9°, 20.6°, and 22.7°. The peaks at 14.9° and 16.9° were assigned to the (1–10) and (110) crystallographic planes of cellulose I structure, respectively. The intense peak at 22.7° corresponded to the (200) plane, indicating a relatively high degree of crystallinity in the cellulose filter paper. The cellulose filbers contained cellulose nanocrystals corresponding to the crystalline domains. The broad reflection centered at approximately 20.6° was associated with partially ordered and amorphous cellulose domains, indicating the coexistence of crystalline and amorphous regions within the filter paper matrix. In addition, the XRD patterns of GO(0.07)/Cel and GO(0.13)/Cel membranes had additional peaks of layered GO structures at 11.4° and 10.4°, corresponding to the *d*-spacing between GO nanosheets of 7.8 Å and 8.5 Å, respectively. It is crucial that both GO(0.07)/Cel and GO(0.13)/Cel membranes contained GO nanochannels with subnanometer *d*-spacing for molecular sieving and ionic conductivity. The pressure-driven formation of the GO membranes resulted in nanochannels with different d spaces that were hydrophilic for water, hydronium, and proton transports.^36–38^

**Fig. 4:**
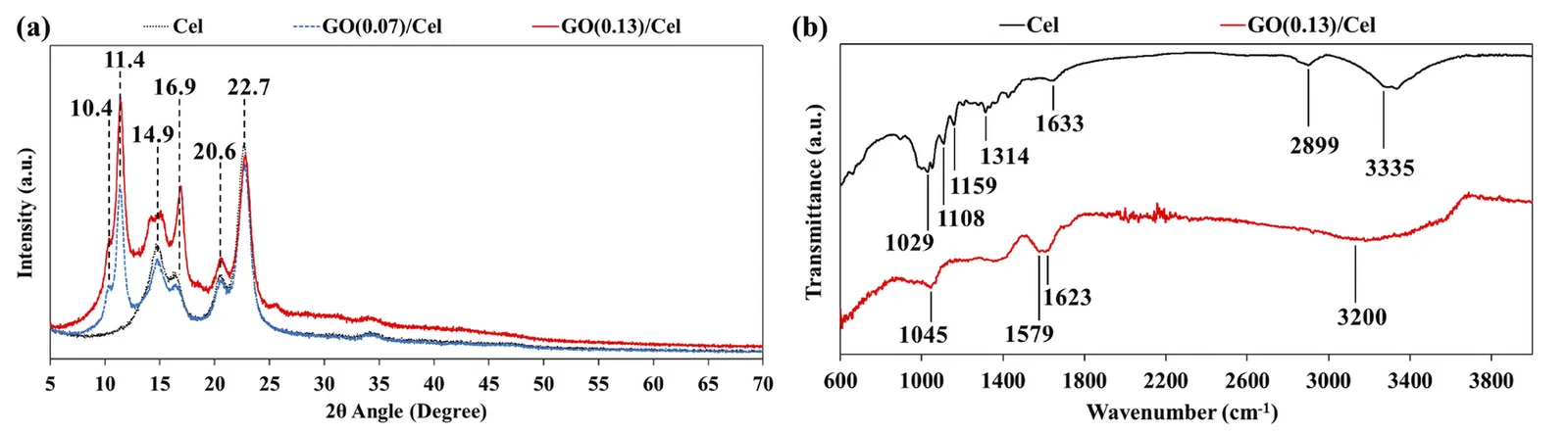
(a) XRD patterns of the Cel substrate, GO(0.07)/Cel membrane, and GO(0.13)/Cel membrane. (b) ATR-FTIR spectra of the Cel substrate and GO(0.13)/Cel membrane.

ATR-FTIR spectrum of the Cel substrate in Fig. 4b exhibited characteristic cellulose absorption bands at 1029 cm^−1^, 1108 cm^−1^, and 1159 cm^−1^. The band at 1029 cm^−1^ is characteristic of C–O stretching vibrations in alcohol groups of cellulose. The band at 1108 cm^−1^ (near 1100 cm^−1^) is assigned to the asymmetric stretching of C–O–C bonds in the glycosidic linkage in the polysaccharide chain of cellulose structure. The peak at 1159 cm^−1^ arises from the asymmetric stretching of the glycosidic ether bridge (C–O–C) connecting glucose units in cellulose chains. The band at 1314 cm^−1^ was attributed to CH_2_ wagging and O–H in-plane bending vibrations associated with crystalline cellulose. Furthermore, the peak at 2899 cm^−1^ was assigned to aliphatic C–H stretching, while the broad band centered at 3335 cm^−1^ corresponded to O–H stretching vibrations involved in hydrogen bonding of cellulose chains and adsorbed water. The small peak at 1633 cm^−1^ originated from the H–O–H bending vibration of adsorbed moisture within the cellulose fibers. Regarding the GO(0.13)/Cel, the ATR-FTIR peaks reflected the functional groups on the surface of the multilayer GO membrane. The bands at 1045 cm^−1^ and 3200 cm^−1^ presented the presence of the oxygen-containing functional groups of epoxide (C–O–C) and hydroxyl (–OH), respectively. The peak at 1579 cm^−1^ was attributed to aromatic π-conjugated domains on GO nanosheets. Besides, the peak at 1623 cm^−1^ was also derived from the vibration of water molecules adsorbed in the layered GO membrane.

The results of nitrogen gas adsorption characterization are presented in Table 2, Fig. 5 and SI Fig. S2. The specific surface area of GO(0.13)/Cel (derived from the BET method) was 3.931 m^2^ g^−1^, which is slightly higher than the value of Cel (3.376 m^2^ g^−1^). The highly compact and nanostructured nature of the GO(0.13) membrane hindered the penetration and adsorption of nitrogen gas during the measurement. The nitrogen adsorption–desorption analysis of the GO membrane revealed differences between the BJH and DFT pore structure evaluations. The BJH method yielded a specific surface area of 3.931 m^2^ g^−1^, a pore volume of 0.011 cm^3^ g^−1^, and an average pore width of 3.793 nm, whereas the DFT model produced lower values of 2.539 m^2^ g^−1^ for surface area and 0.008 cm^3^ g^−1^ for pore volume, with a comparable pore width of 3.775 nm. The close agreement in pore width obtained by both methods confirms that the GO(0.13)/Cel material contained mesopores with diameters near 3.79 nm. It is notable that GO(0.13)/Cel had a considerable proportion of pore size of 1.45 nm (see inset in Fig. 5b) as calculated by BJH method. The BJH analysis, which is based on Kelvin equation for capillary condensation, may assign interparticle voids or interlayer spaces to the pore size distribution. Besides, the DFT analysis incorporates a molecular statistical model of gas adsorption and pore filling, leading to a representation of confined pore network in the material. Both BJH and DFT calculations identified a mesoporous architecture, providing general assessment of cumulative pore volumes and pore size distributions of the GO(0.13)/Cel membrane.

**Table 2:** Gas adsorption analysis of specific surface areas and pore structures of Cel and GO(0.13)/Cel membranes. The values of Cel are referenced and cited from our previous paper.^31^

Calculation method	Cel membrane	GO(0.13)/Cel membrane
BET surface area (m^2^ g^−1^)	3.376	3.931
BJH pore volume (cm^3^ g^−1^)	0.008	0.011
BJH pore width (nm)	2.721	3.793
DFT surface area (m^2^ g^−1^)	2.449	2.539
DFT pore volume (cm^3^ g^−1^)	0.006	0.008
DFT pore width (nm)	6.079	3.775

**Fig. 5:**
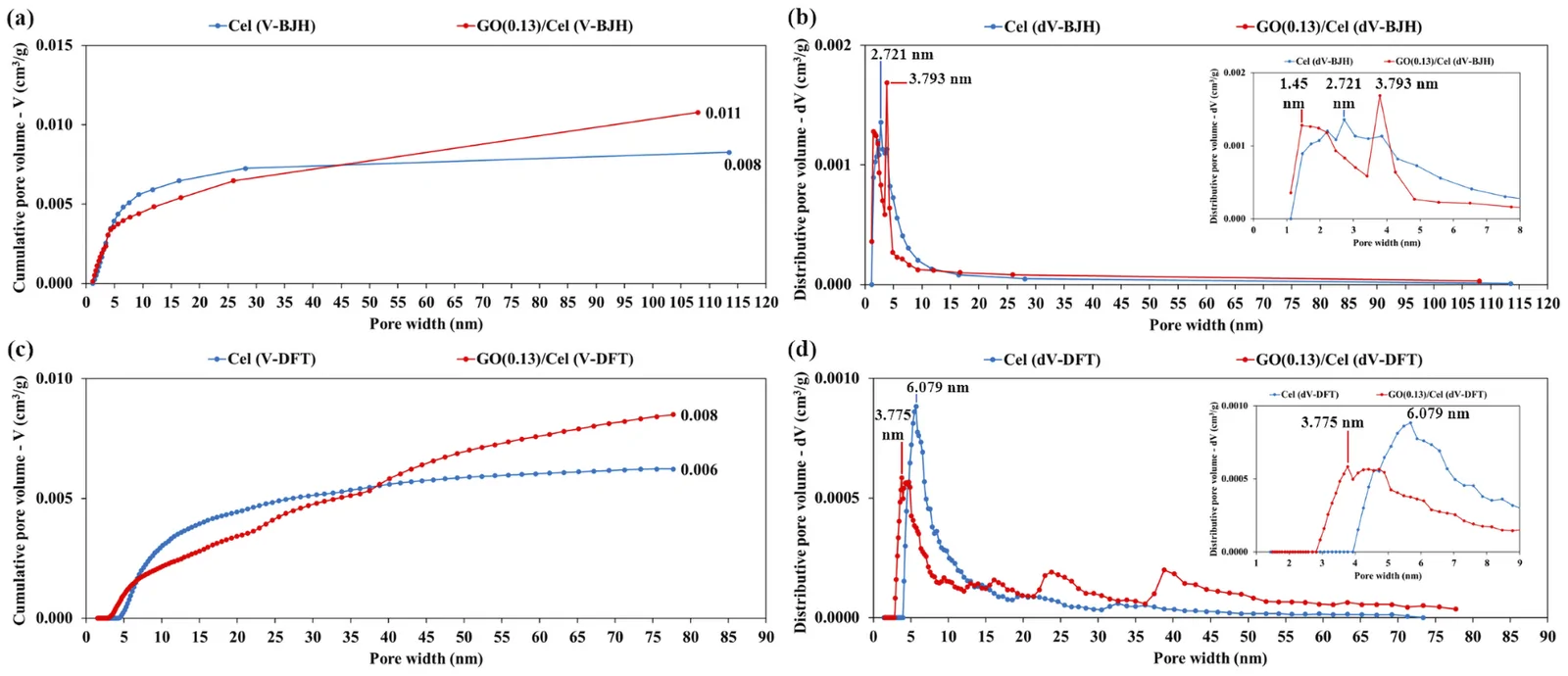
Cumulative pore volume (a) and pore volume distribution (b) of the Cel paper and GO(0.13)/Cel membrane derived from the BJH calculation method. Cumulative pore volume (c) and pore volume distribution (d) of Cel paper and GO(0.13)/Cel membrane calculated from DFT modeling. Insets are the focuses on microporous volumes.

The wettability of Cel paper and GO(0.13)/Cel membranes exhibited distinct characteristics owing to their different surface structures and water absorption behaviors. For the Cel filter paper, the deposited water droplet rapidly penetrated into the porous fibrous network, making the contact angle difficult to measure accurately and indicating a highly hydrophilic and absorbent surface (Fig. 6a). This behavior is attributed to the abundance of hydroxyl groups on cellulose chains combined with the interconnected microscale pores that promote capillary-driven water uptake. In contrast, the GO membrane exhibited a measurable static water contact angle of 32.52°, determined at 20 °C using a 3.95 µL water droplet (Fig. 6b). Although the GO membrane was also hydrophilic due to the presence of oxygen-containing functional groups such as hydroxyl, epoxy, and carboxyl groups, the relatively compact layered structure of the membrane limited immediate water penetration, allowing the droplet to remain on the surface during measurement. Therefore, while both materials possessed hydrophilic surfaces, the cellulose filter paper demonstrated superior wettability characterized by instantaneous water absorption, whereas the GO membrane exhibits moderate wetting with a specific contact angle, reflecting the combined effects of surface chemistry and membrane morphology.

**Fig. 6:**
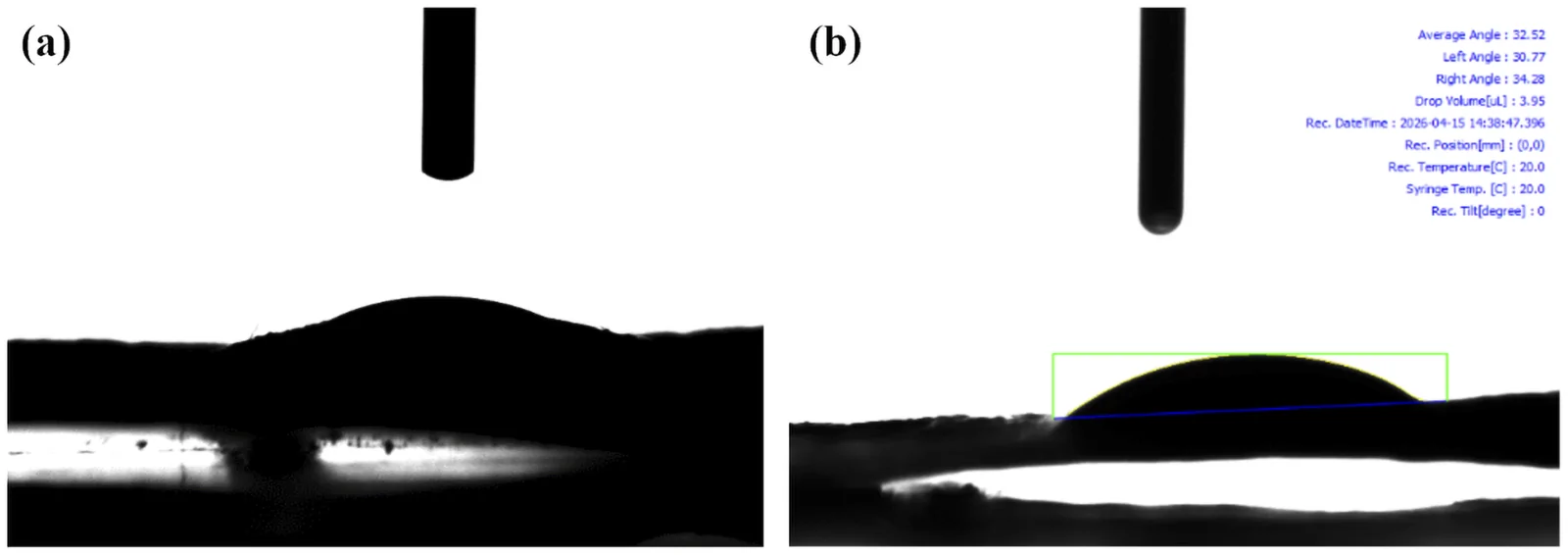
Contact angle measurements of the Cel substrate (a) and GO(0.13)/Cel membrane (b).

### Organic dye removal efficiencies of multilayered-graphene-oxide/cellulose membranes

Direct-flow water-filtration experiments were set up as described in Fig. 7a–c. The photograph in Fig. 7a shows the top view of a circular Cel(0.13)/Cel membrane fixed in a glass funnel containing 300 mL water. The filtration system was equipped with a high-performance vacuum pump to create a transmembrane pressure of 1 bar. The GO/Cel membranes were typically tested for the filtration of pure water, methylene blue solution 20 ppm (MB20, Fig. 7b), and rhodamine B solution 20 ppm (RhB20, Fig. 7c). The chart of water fluxes in Fig. 7d shows the ultrafast water permeance of GO(0.07)/Cel (959.8 L m^−2^ h^−1^ bar^−1^ for water and 685.6 L m^−2^ h^−1^ bar^−1^ for MB20). The GO(0.07)/Cel membrane also attained relatively high efficiencies of dye removal, specifically 92.1% for MB20, 77.1% for MB40, and 71.8% for RhB20 (Fig. 7e). After depositing the secondary GO layer, the obtained GO(0.13)/Cel membrane presented lower water permeances (240.5 L m^−2^ h^−1^ bar^−1^ for water and 158.4 L m^−2^ h^−1^ bar^−1^ for MB20) and higher efficiencies of dye removal (99.9% for MB20 and 96.7% for RhB20). The membrane structure of GO(0.07)/Cel containing microscale cracks was significantly improved with the secondary GO layer, resulting in a GO(0.13)/Cel membrane with high dye removal efficiencies and relatively high water permance (in the range of 147.4–240.5 L m^−2^ h^−1^ bar^−1^).

**Fig. 7:**
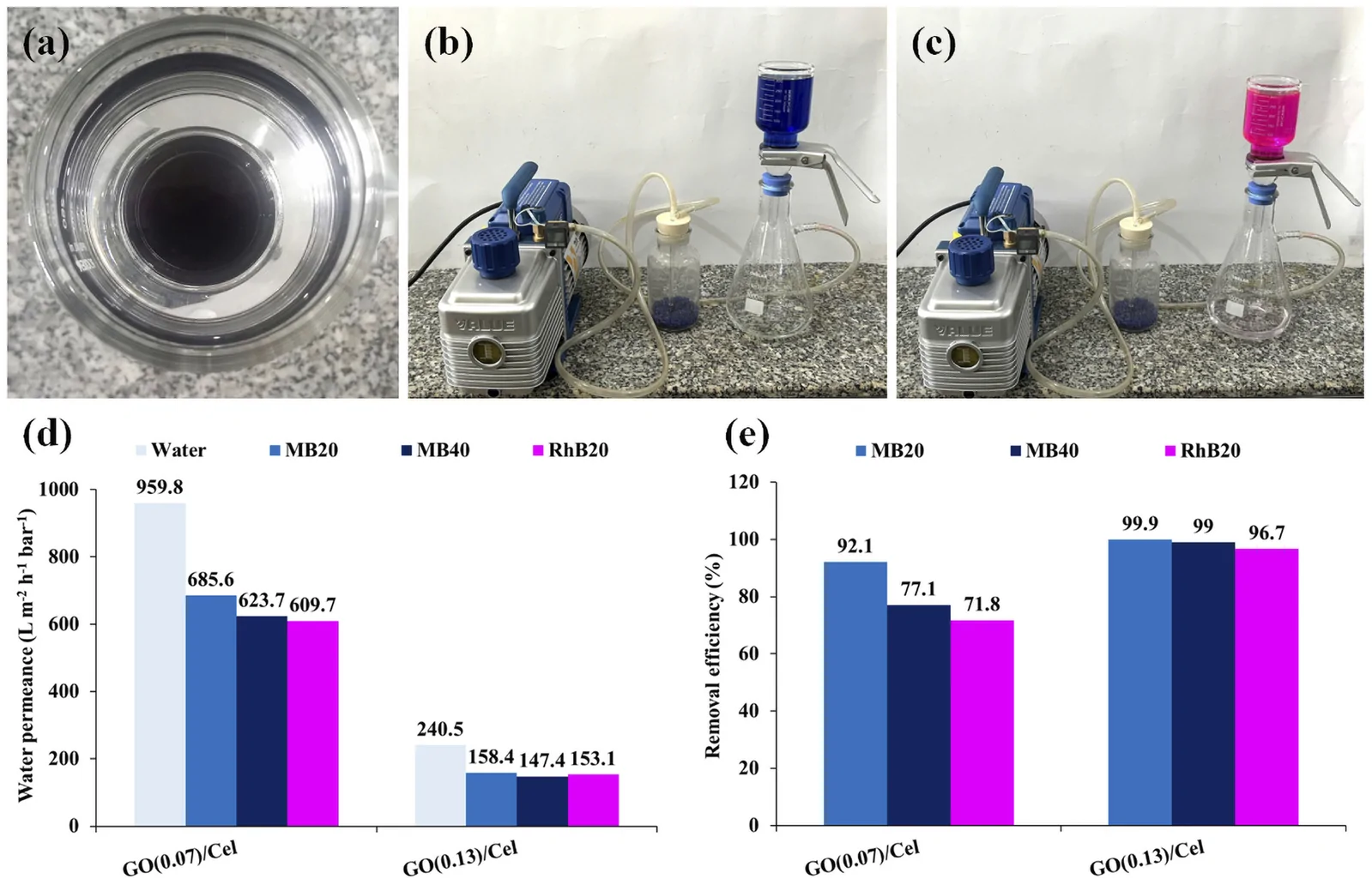
(a) Top-view photograph of a GO(0.13)/Cel membrane clamped in a glass funnel filled with water. Experimental setups of direct-flow filtrations of MB solution 20 ppm (b) and RhB solution 20 ppm (c). (d) Chart of water permeances through GO(0.07)/Cel and GO(0.13)/Cel membranes under a transmembrane pressure of 1 bar. (e) Chart of pollutant removal efficiencies of GO(0.07)/Cel and GO(0.13)/Cel membranes.

The high performance of GO(0.13)/Cel is quite good for practical water filtration applications compared with the scientific references in Table 3.^39–46^ Similar performances of water filtration were reported in scientific literature, including reduced graphene oxide membrane (S-rGO),^41^ GO/halloysite-nanotubes membrane,^42^ and GO/NGO/Mxene membrane.^43^ While the graphene-based membranes in many previous studies were prepared using microfiltration membranes,^5–10,41–44^ the GO(0.13)/Cel membrane in this study was synthesized using a regular cellulose paper with the advantages of inexpensiveness, biodegradability, and ultrafast water permeance. The drawback of the large pore size (15–20 µm) of the Cel filter paper was solved by the vacuum filtration depositions of primary and secondary GO layers. The primary GO layer was constructed by GO aggregates in the dispersion with the higher concentration of 240 ppm. As the primary GO layer on the porous and flexible Cel substrate was fractured, microscopic cracks were observed on the GO(0.07)/Cel membrane (SEM image in Fig. 2e). Thus, the secondary GO layer was constructed on the relatively flat surface of GO(0.07)/Cel membrane using the GO dispersion with the lower concentration of 60 ppm. Although the vacuum filtration deposition of the dilute GO dispersion was slower, the secondary GO layer was better nanostructured through the gradual deposition of GO nanosheets.

**Table 3:** Summary of experimental results of water filtration membranes, water permeances, and removal efficiencies

Membrane materials	Pollutant	Water permeance (L m^−2^ h^−1^ bar^−1^)	Removal efficiency (%)	References
Cellulose nanofiber membrane	Methylene blue	306	96.5	39
Cellulose ultrafiltration membrane	Bovine serum albumin protein	188	95.2	40
Reduced graphene oxide membrane (S-rGO)	Methylene blue mixed with microplastics	242.6	99.9	41
GO/halloysite-nanotubes membrane	Methylene blue	206.7	98.5	42
GO/NGO/Mxene membrane	Methylene blue	136.68	99.99	43
ZnO/RGO membrane	Methylene blue	225	98.1	44
GO/SiO_2_ membrane	Rhodamine B	229.15	98.3	45
GO composite membrane	Congo red	240	95	46
GO(0.07)/Cel membrane	Methylene blue	623.7–685.6	77.1–92.1	This work
GO(0.13)/Cel membrane	Methylene blue	147. 4–158.4	99–99.9	This work

In addition, the long-term stability of the GO(0.13)/Cel membrane was investigated by testing and characterizing the GO(0.13)/Cel membrane after 15-days of immersion in water (denoted as GO(0.13)/Cel-15d). Fig. 8a shows the top-view picture of the GO(0.13)/Cel-15d membrane. SEM images in Fig. 8b and c show that the membrane surface was uniformly covered by GO nanosheets without considerable structural degradation. Water filtration experiments with MB20 and MB40 solutions revealed that the GO(0.13)/Cel-15d membrane maintained high performance, with water permeances of approximately 200 L m^−2^ h^−1^ bar^−1^ and dye removal efficiencies above 90% (Fig. 8d and e). The π–π stacking and crosslinking of GO nanosheets accounted for the membrane stability and filtration performance. In addition, retained organic molecules would also contribute to interconnections in the GO membrane.^38^ Cross-sectional SEM images in Fig. 9 further clarify the formation and structure of GO(0.07)/Cel, GO(0.13)/Cel, and GO(0.13)/Cel-15d membranes. Fig. 9a shows the cross-section SEM of GO(0.07)/Cel, which reveals that the primary GO layer had microscale cracks and different thicknesses from 15 µm to 30 µm on the Cel substrate. The assembly of GO nanosheets in the primary GO layer was focused in Fig. 9b. After the formation of the secondary GO layer, the cross-section SEM images of GO(0.13)/Cel (Fig. 9c and d) exhibited a compact layered structure and uniform thickness (approximately 30 µm) of the GO multilayer membrane. The membrane thickness of ∼30 µm was comparable to that of the primary GO layer at some positions, suggesting that the vacuum filtration deposition of the secondary GO layer made the multilayer GO membrane more compact and layered. Therefore, the GO(0.13) membrane had better nanostructured channels for water/proton transport without significant microscale cracks. Fig. 9e and f show the cross-section SEM images, which reveal that the GO(0.13)/Cel-15d maintained a compact layered structure on the cellulose filter support, which was responsible for the good performance of the GO(0.13)/Cel-15d membrane.

**Fig. 8:**
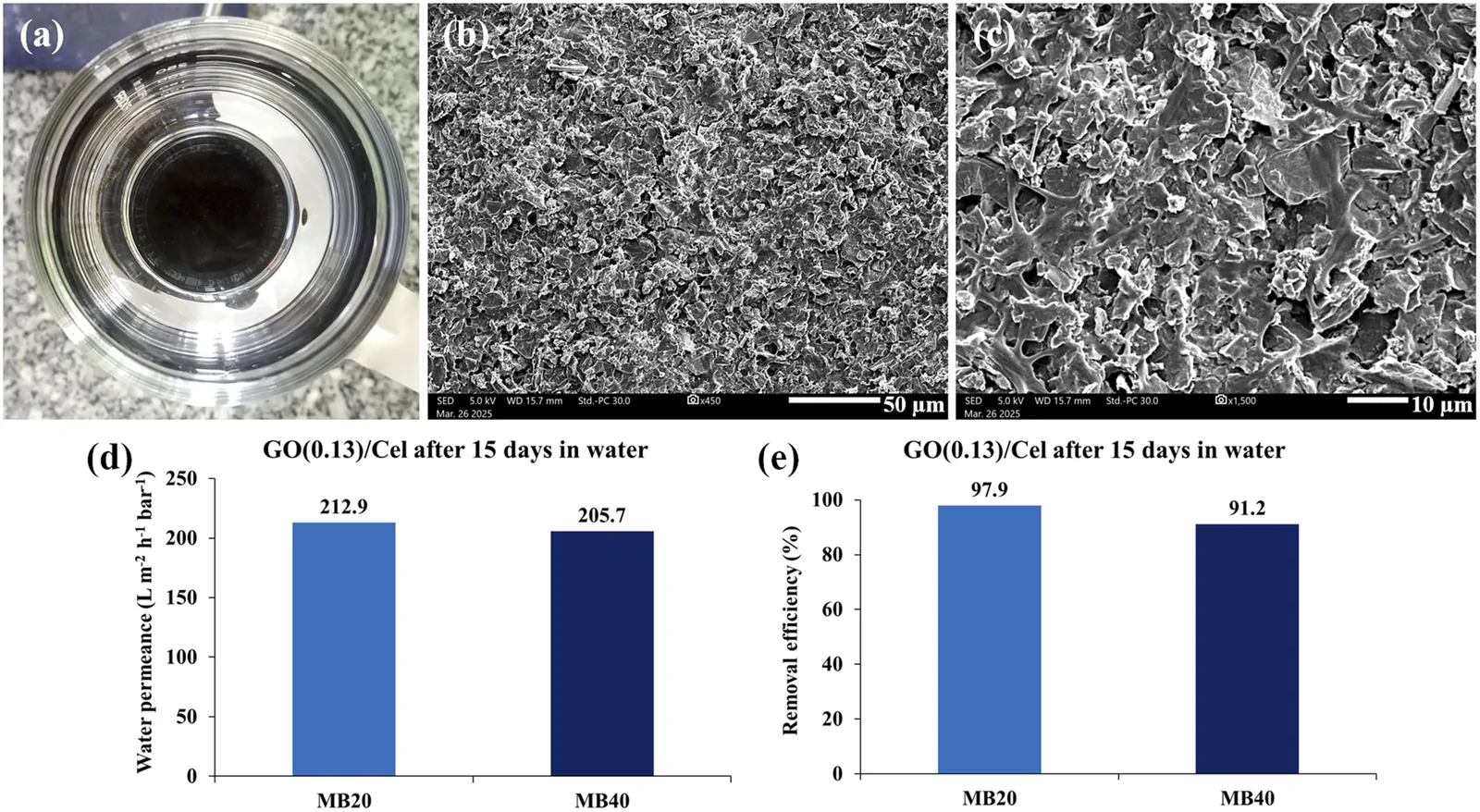
(a) GO(0.13)/Cel membrane after water filtration and immersion in water for 15 days (GO(0.13)/Cel-15d). Top-view SEM images of the GO(0.13)/Cel-15d membrane with scale bars of 50 µm (b) and 10 µm (c). Charts of water permeances (d) and pollutant removal efficiencies (e) of the GO(0.13)/Cel-15d membrane.

**Fig. 9:**
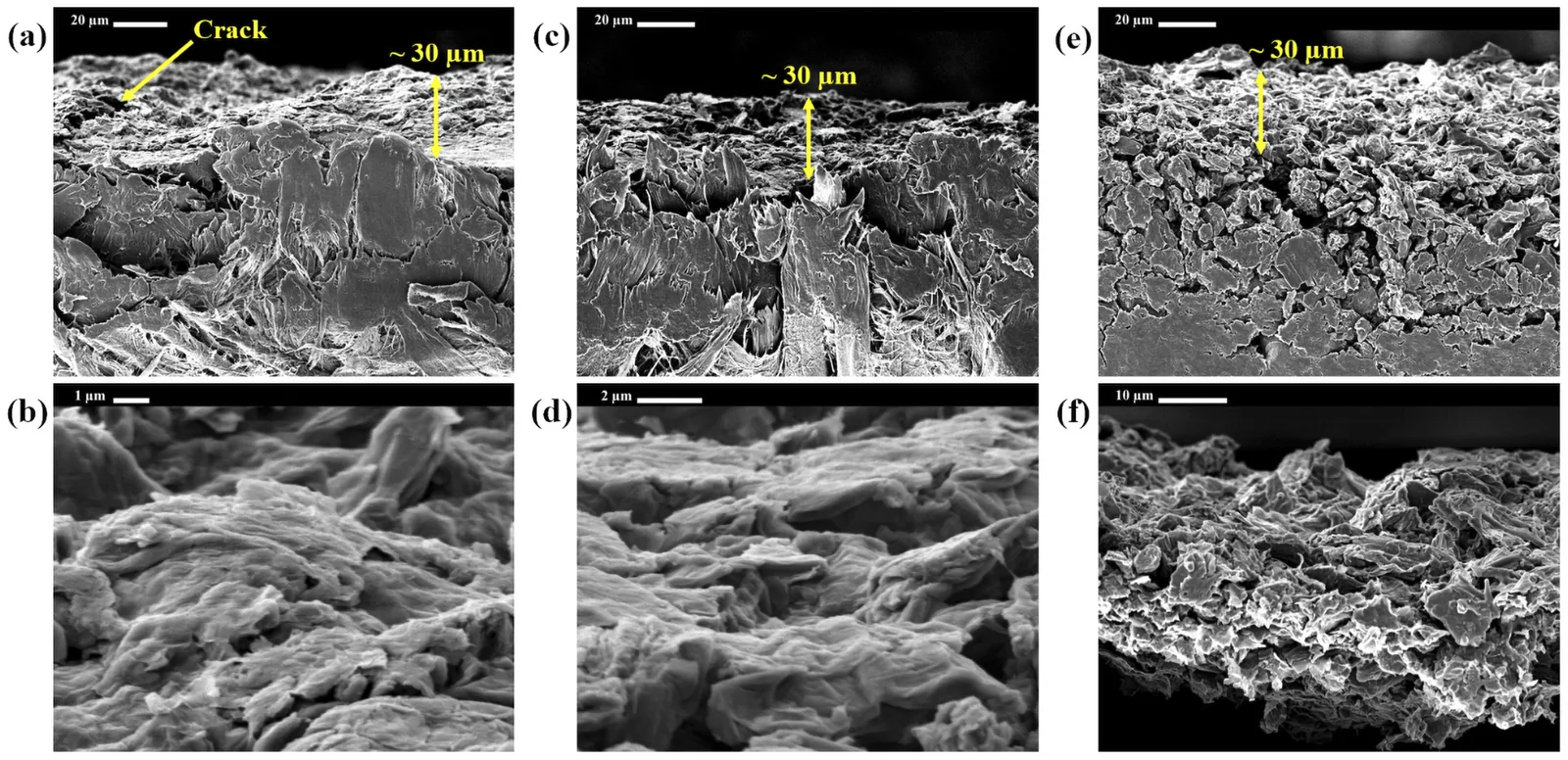
Cross-sectional SEM images of the GO(0.07)/Cel with scale bars of 20 µm (a) and 1 µm (b). Cross-sectional SEM images of the GO(0.13)/Cel membrane with scale bars of 20 µm (c) and 2 µm (d). Cross-sectional SEM images of the GO(0.13)/Cel-15d membrane with scale bars of 20 µm (e) and 10 µm (f).

### Synthetic seawater desalination using cellulose/multilayered-graphene-oxide/cellulose membranes

With good performance in the separation of organic dye molecules, the GO(0.13)/Cel membrane was tested in the direct-flow filtration of synthetic seawater (NaCl solution 2.8%). To maintain the GO membrane structure in many cycles of filtration and washing, another Cel paper was placed on the top of GO(0.13)/Cel, forming a sandwich structure of Cel/GO(0.13)/Cel (as depicted in Fig. 10a). The sandwich structure made the multilayer GO membrane more stable during the filtration and washing processes. The water permeances and NaCl concentrations are plotted in Fig. 10b. In the first three cycles, the NaCl concentration in the synthetic seawater decreased by 40% (from 28 000 ppm to 16 800 ppm). In the next three cycles, a decline of 22.5% was recorded (from 16 800 ppm to 10 500 ppm). The decrease in the NaCl concentration was slower, from 10 500 ppm in the 6th cycle to 8936 ppm in the 12th cycle, which was a decline of ∼5.6% in six filtration times. It is noteworthy that after 12 filtration cycles, the synthetic seawater was desalinated to obtain a saline solution with a NaCl concentration of nearly 0.9%. Saline solution 0.9%, also called physiological saline, is useful for biomedical and clinical applications, such as wound cleansing, eye and nasal irrigations, and biomedical rinsing. In addition, the water permeances gradually declined from 248.3 L m^−2^ h^−1^ bar^−1^ in the first filtration to 220.4 L m^−2^ h^−1^ bar^−1^ in the 12th filtration due to the fouling of NaCl salts in the GO membrane. Although the sandwich membrane Cel/GO(0.13)/Cel was washed with clean water after every filtration, the multilayer membrane was saturated with NaCl fouling, resulting in a gradual decrease in the desalination performance in later cycles. In general, the multilayer membrane is quite effective for desalination in the first six filtration cycles (removal of 62.5% NaCl in the synthetic seawater) and would be potential to convert seawater into physiological saline 0.9%.

**Fig. 10:**
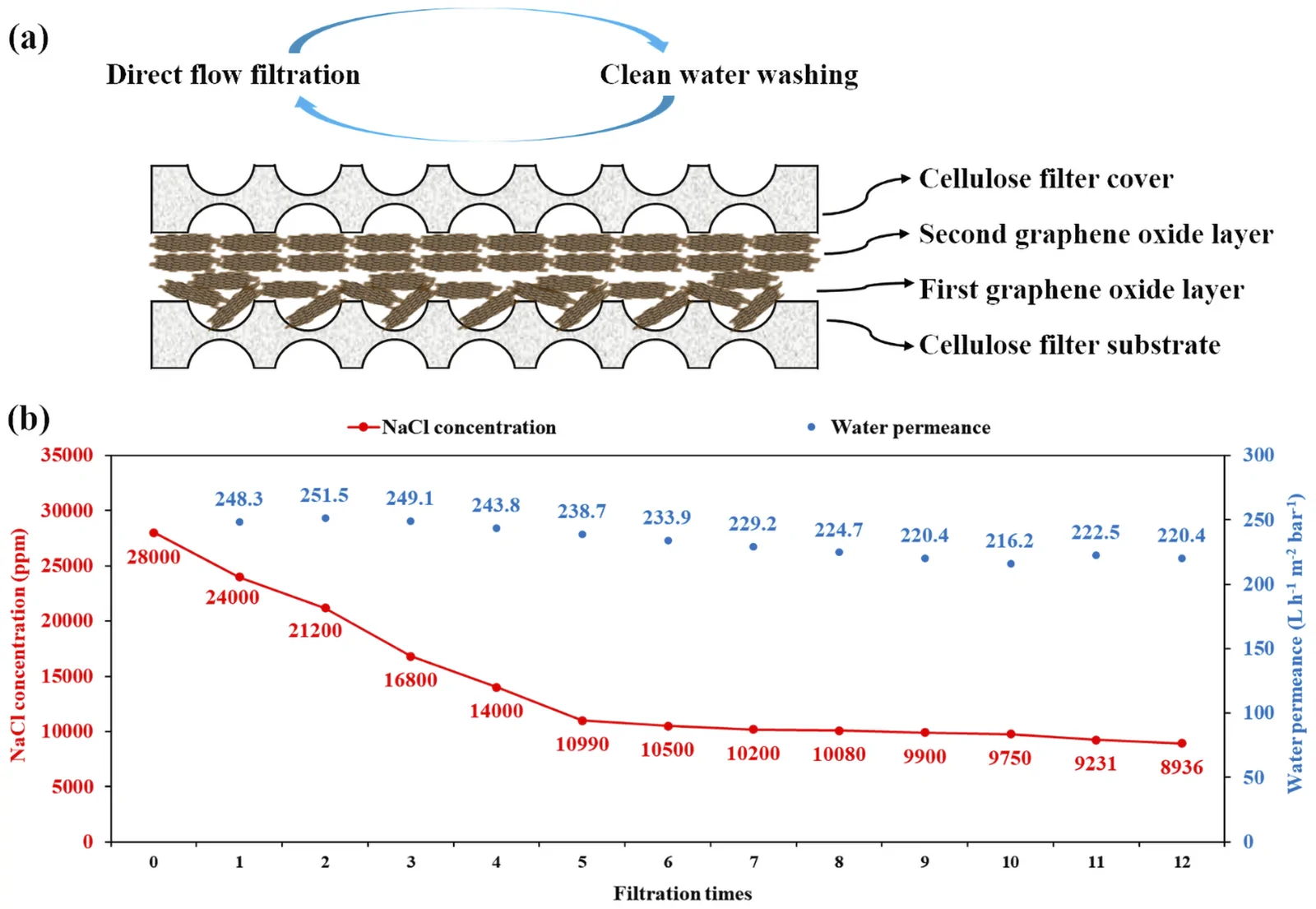
(a) Illustration of the multilayered GO membrane on a Cel substrate and covered by a Cel paper, resulting in a sandwich membrane structure for repeatitive cycles of water filtration and water washing. (b) Chart presentation of NaCl concentration and water permeance with respect to the number of filtration cycles.

### Measurement of tensile properties of graphene-oxide/cellulose and sandwich membranes

Tensile testing of GO(0.07)/Cel and GO(0.13)/Cel membranes was performed to evaluate the mechanical properties, including tensile strength, elastic modulus, elongation at break, and energy to break (Fig. 11, SI Table S1 and Fig. S3–S6). According to Fig. 11a, the increasing tensile strength from 8.55 MPa of Cel to 8.63 MPa of GO(0.07)/Cel and 10.35 MPa of GO(0.13)/Cel revealed the important role of the nanostructured coatings in strengthening the cellulose films as the tensile strength of freestanding GO membrane was measured to be 59.17 MPa.^31^ The elastic modulus, which is the resistance to elastic deformation, was greatly enhanced from 679.14 MPa of Cel to 1081.96 MPa of GO(0.07)/Cel and 1348.24 MPa of GO(0.13)/Cel. Particularly, GO(0.13)/Cel exhibited the significant increases of 19.93% in tensile strength and 24.61% in elastic modulus compared to those of GO(0.07)/Cel. As presented in Fig. 11b, the tensile elongation and break energy of GO(0.13)/Cel, corresponding to the membrane ductility and the total energy absorbed before fracture, were respectively 39.61% and 67.86% higher than those of GO(0.07)/Cel. As a result, GO(0.13)/Cel membrane is considered as much more compact and crosslinked than GO(0.07)/Cel membrane. Regarding the sandwich structures of GO membranes between Cel papers, the tensile strengths of Cel/GO(0.07)/Cel (8.11 MPa) and Cel/GO(0.13)/Cel (9.36 MPa) were slightly lower than those of GO(0.07)/Cel and GO(0.13)/Cel because the proportions of GO layers in the sandwich structures were smaller. However, it was found that the sandwich structures provided higher values of elongation and toughness. Specifically, the tensile energy of Cel/GO(0.07)/Cel (2.01 kgf.mm) had the increases of 8.06%, 113.83%, and 258.93% in comparison with those of Cel/GO(0.07)/Cel (1.86 kgf.mm), GO(0.13)/Cel (0.94 kgf.mm), and GO(0.07)/Cel (0.56 kgf.mm) respectively. The additional Cel layer made the sandwich structure more flexible to obtain the higher elongation and larger energy to break. In general, the sandwich structure of Cel/GO(0.13)/Cel is stronger and tougher than Cel/GO(0.07)/Cel owing to the better nanostructured multilayer GO(0.13) membrane.

**Fig. 11:**
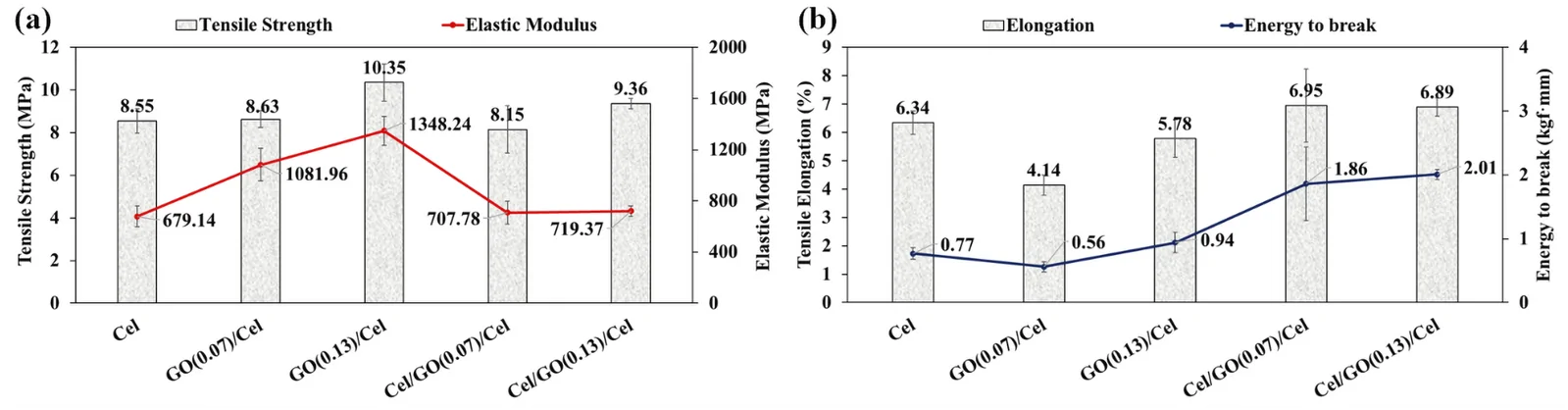
Mechanical properties of multilayer GO/Cel and sandwich Cel/GO/Cel membranes. (a) Tensile strength and elastic modulus. (b) Tensile elongation and energy to break.

### Measurement of proton conductivities of cellulose/multilayered-graphene-oxide/cellulose membranes

In the aspect of proton conduction, cellulose materials have been investigated to exhibit considerable proton conductivity under anhydrous conditions at elevated temperatures.^47^ Recent researches on GO membranes have suggested that hydrophilic GO nanochannels in can be engineered to provide nanoconfined pathways for fast water/proton transport.^27,48,49^ In this study, sandwich structures of Cel/Cel, Cel/GO(0.07)/Cel, and Cel/GO(0.13)/Cel membranes were prepared for EIS measurements of membrane resistances and proton conductivities. Proton transport in the separation membranes occurs through two distinct mechanistic pathways: vehicular proton diffusion and Grotthuss proton hopping. Particularly, protons hopping along hydrogen-bond networks in the nanochannels of GO membranes is considered as the highway for fast proton transport as well as higher proton conductivity. Previous study in scientfic literature revealed that the proton transport mechanisms in GO nanochannels are fundamentally governed by the water content and membrane hydration state.^27^ At approximately two layers of water molecules, a complete hydrogen-bond network formed in the GO nanochannel significantly improves hydrogen-bond coordination for fast proton hopping in the in-plane direction, leading to high total conductivity. At higher water contents, the conduction process is dominated by the diffusion of hydronium molecules rather than proton hopping. Protons diffuse through the membrane in the form of hydronium ions dissolved in water and spread in both in-plane and out-of-plane directions inside the GO nanochannel. Therefore, GO membranes nanostructured for water-mediated proton hopping are important for applications requiring fast proton transport, such as proton exchange membrane fuel cells, proton exchange membrane electrolyzers and electrochemical sensors.

The EIS spectra, equivalent circuits, fitting curves, and resistance values of Cel/Cel, Cel/GO (0.07)/Cel, and Cel/GO (0.13)/Cel membranes are shown in Fig. 12a–c, respectively. Proton conductivities were calculated from membrane resistances in the condition of 25 °C and 100% RH. According to the results in Fig. 12d, the proton conductivity increased from 1.08 mS cm^−1^ for Cel/Cel to 9.56 mS cm^−1^ for Cel/GO(0.07)/Cel due to the presence of primary GO membrane in Cel/GO(0.07)/Cel membrane. Proton transport pathways through Cel/GO(0.07)/Cel membrane are illustrated in Fig. 12e. It is explained that hydronium diffusion mechanism was dominant in the large pore structure of Cel papers, and hopping mechanism occurred for faster proton transport in nanochannels of GO(0.07) membrane. The XRD pattern in Fig. 4a shows that the GO(0.07) layer contained nanochannels with a *d*-spacing of 8.5 Å, which was attributed to approximately one water layer in the nanochannels.^27^ GO nanochannels containing one water layer were highways for fast proton hopping mechanism. However, the top-view SEM image in Fig. 2e and the cross-sectional SEM image in Fig. 9a showed microscale cracks and varied thicknesses of the GO(0.07) layer, providing spaces for hydronium diffusion. Therefore, proton transport through the GO(0.07) layer included proton diffusion and proton hopping pathways, resulting in a considerable increase in the total proton conductivity. Regarding the multilayer membrane Cel/GO(0.13)/Cel, a high proton conductivity of 17.59 mS cm^−1^ was achieved thanks to the better nanostructure of the GO(0.13) membrane. The XRD pattern (Fig. 4a) and ATR-FTIR spectrum (Fig. 4b) of GO(0.13)/Cel also indicated that the multilayer GO(0.13) membrane contained nanochannels with hydrophilic functional groups (FTIR peaks of epoxide and hydroxyl groups) and one water layer inside (*d*-spacing of 8.5 Å). SEM images in Fig. 2h, i, 8b, c, 9c and e presented that GO(0.13) membrane had uniform surface, layered structure and compact thickness (∼ 30 µm). The better nanostructured GO(0.13) membrane provided more hydrophilic nanochannel highways for the proton hopping mechanism, as described in Fig. 12f. Proton transport pathways through hydrogen-bond networks in GO(0.13) membrane were more cohesive, leading to the high proton conductivity of 17.59 mS cm^−1^.

**Fig. 12:**
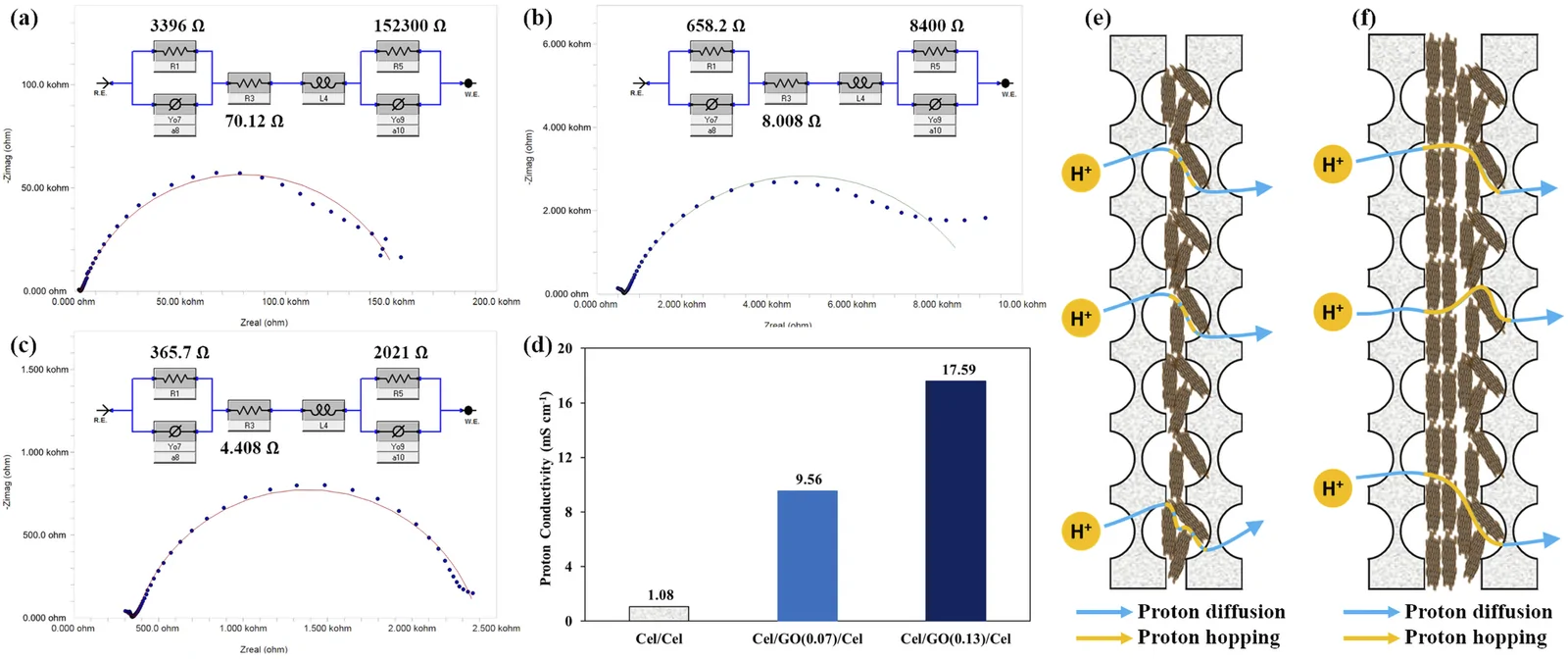
Nyquist plots of EIS spectra of a Cel/Cel membrane (a), a Cel/GO(0.07)/Cel membrane (b), and a Cel/GO(0.13)/Cel membrane (c), with the inset diagrams of equivalent fuel cell circuits. Curve autofitting and membrane resistance values were obtained using Gamry Echem Analyst software. (d) Chart of proton conductivities of Cel/Cel, Cel/GO(0.07)/Cel, and Cel/GO(0.13)/Cel membranes at relative humidity of 100% and room temperature of 25 °C. Descriptions of proton transport pathways *via* diffusion and hopping mechanisms through Cel/GO(0.07)/Cel (e) and Cel/GO(0.13)/Cel membranes (f).

In comparison with other studies on proton-conducting membranes (Table 4), the proton conductivities of commercial Nafion 117 were 6 mS cm^−1^ at 25 °C and 80% RH, 16 mS cm^−1^ at 60 °C and 80% RH, and reached 66 mS cm^−1^ at 60 °C and 100% RH.^33^ M. R. Karim *et al.* demonstrated that the proton conductivty of a single GO nanosheet was 5.7 mS cm^−1^ at 25 °C and 95% RH.^52^ In the paper by L. Zhang *et al.*, layered GO membranes (thickness of 60 µm and water content of ∼ 41%) were analyzed by EIS measurements to show the maximum proton conductivity of 33 mS cm^−1^.^27^ However, the overall proton conductivity decreased to 6 mS cm^−1^ in the sandwich structure of Nafion/GO/Nafion due to the resistances of interfacial contacts between the Nafion and GO layers. Therefore, in this work, the proton conductivities of 9.56 mS cm^−1^ for Cel/GO(0.07)/Cel and 17.59 mS cm^−1^ for Cel/GO(0.13)/Cel are relatively high in comparison with Nafion membranes and other sandwich structures reported in the scientific literature. Moreover, membrane resistances were measured at higher temperatures for analyzing the temperature-dependent proton conductivities (Fig. 13a, SI Table S2 and S3). The plot of linear regression in Fig. 13b exhibited linear equations with R-squared values and specific slopes for calculation of activation energies of the sandwich membranes.

**Table 4:** Summary of proton-conducting membranes and proton conductivities under different measurement conditions

Proton-conducting membranes	Proton conductivity (mS cm^−1^)	Measurement conditions	References
Nafion 117 membrane pretreated with 1 M H_2_SO_4_ solution	6	25 °C, 80% RH, contact pressure of 1000 KPa	33
	16	60 °C, 80% RH, contact pressure of 1000 KPa	33
	66	60 °C, 100% RH, contact pressure of 1000 KPa	33
Silanol-containing nanosilica	2.58	30 °C, 98% RH	50
Functionalized silica	10	40 °C, 100% RH	51
GO nanosheet	5.7	25 °C, 95% RH	52
GO membrane	33	GO membrane thickness of 60 µm, water content of 0.7 g H_2_O/g GO	27
Nafion/GO/Nafion membrane	6		
Cel/Cel	1.08–4.82	25–45 °C, 100% RH	This work
Cel/GO(0.07)/Cel	9.56–28.62		
Cel/GO(0.13)/Cel	17.59–44.83		

**Fig. 13:**
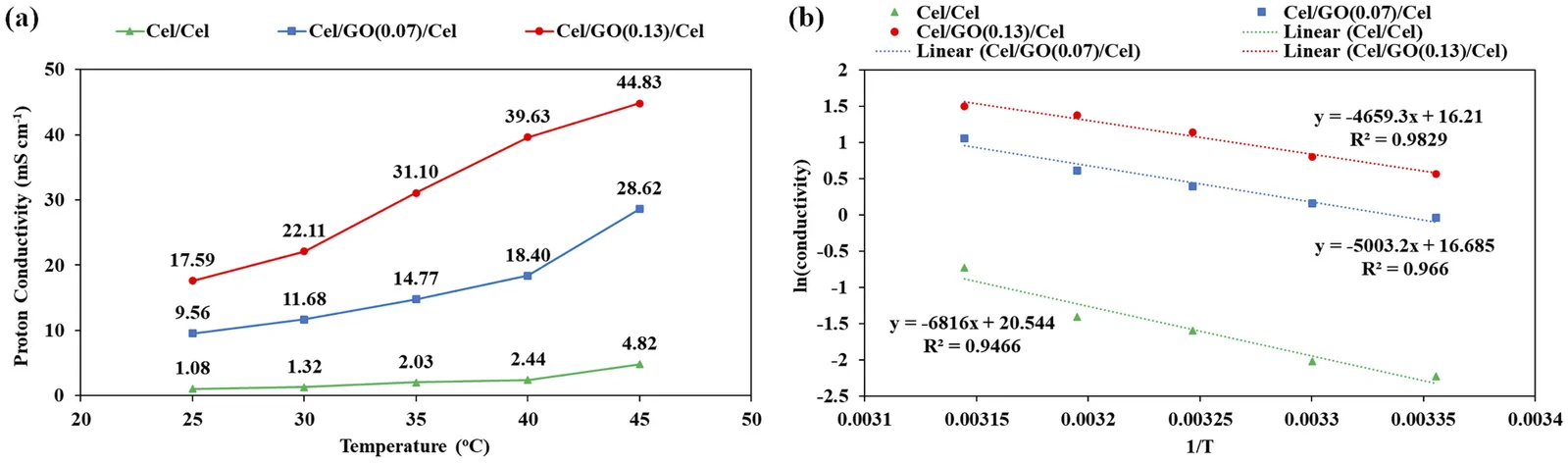
(a) Temperature-dependent conductivities of Cel/Cel, Cel/GO(0.07)/Cel, and Cel/GO(0.13)/Cel membranes at 100% RH and different temperatures. (b) Linear plot of ln(conductivity) and 1/T for calculating activation energies of Cel/Cel (56.67 KJ mol^−1^), Cel/GO(0.07)/Cel (41.6 KJ mol^−1^), and Cel/GO(0.13)/Cel membranes (38.74 KJ mol^−1^) using the Arrhenius equation.

As a result, the activation energy of Cel/Cel membrane (56.67 KJ mol^−1^) implied a relatively high barrier for proton conduction. Cel/GO(0.07)/Cel membrane presented a lower activation energy (41.6 KJ mol^−1^) for facile proton transport. Especially, Cel/GO(0.13)/Cel membrane showed the significant increase of proton conductivity from 17.59 mS cm^−1^ at 25 °C to 44.83 mS cm^−1^ at 45 °C, leading to a smaller activation energy (38.74 KJ mol^−1^) as well as the lowest energetic barrier for proton conduction. Although the activation energy barrier of Cel/GO(0.13)/Cel was only 6.9% lower than that of Cel/GO(0.07)/Cel, the significantly higher conductivities of Cel/GO(0.13)/Cel are attributed to the compact nanostructure of GO(0.13) membrane that provided more nanochannel highways for fast proton transport. As-synthesized GO membranes have hydrogen-bond networks and good interfacial contact with hydrophilic Cel papers for compatible proton transfer. Generally, the sandwich structure of Cel/GO(0.13)/Cel is a novel assembly for proton-conducting membranes in eco-friendly energy devices.

## Conclusions

Advances in GO membrane synthesis have been developed for the preparation of multilayer GO membranes on Cel filter paper using the vacuum filtration deposition method. As the Cel substrate had the large pore size of 15–20 µm, it is necessary to form the primary GO layer using the GO dispersion 240 ppm. To cover microscale fractures in the primary GO layer, vacuum filtration of GO dispersion 60 ppm was carried out for the deposition of the secondary GO layer, producing the multilayer GO(0.13)/Cel membrane. XRD, ATR-FTIR, SEM, EDS, gas adsorption, and contact angle characterizations showed that the GO(0.13)/Cel contained functional groups and hydrophilic nanochannels for water and proton transports. Water filtration experiments demonstrated the high performance of the GO(0.13)/Cel, including the water permeance of 147.4–240.5 L m^−2^ h^−1^ bar^−1^ and the dye removal efficiency of 96.7–99.9%. The performance and structure of GO(0.13)/Cel were maintained after 15 days in water, suggesting the membrane stability for long-term operation. The sandwich structure of Cel/GO(0.13)/Cel presented the higher tensile strength and break energy for mechanical stability, enabling the desalination of NaCl solution 2.8% to produce a physiological saline 0.9% after 12 filtration cycles. EIS analyses at 100% RH and 25–45 °C temperatures revealed the significant rises of proton conductivities from 1.08–4.82 mS cm^−1^ for Cel/Cel membrane to 9.56–28.62 mS cm^−1^ for Cel/GO(0.07)/Cel membrane, and 17.59–44.83 mS cm^−1^ for Cel/GO(0.13)/Cel membrane. The energetic barriers for proton conduction decreased from 56.67 KJ mol^−1^ for Cel/Cel to 41.6 KJ mol^−1^ for Cel/GO(0.07)/Cel, and further to 38.74 KJ mol^−1^ for Cel/GO(0.13)/Cel. While microscale cracks and voids in the GO(0.07) membrane limited its proton conductivity and activation energy, the compact multilayer GO(0.13) membrane contained superior nanostructured nanochannel highways for fast proton transport with lower activation energy barrier. The Cel/GO(0.13)/Cel membrane represents a novel multilayer structure for multifunctional applications in water purification systems and proton exchange membrane devices.

## Author contributions

Hon Nhien Le: conceptualization; methodology; investigation; formal analysis; data curation; visualization; funding acquisition; project administration; writing – original draft; and writing – review & editing. Ngoc Quang Tran: investigation; formal analysis; data curation. Nang An Vu: investigation; resources; data curation. Lam Nhu Pham: experimental synthesis; formal analysis; investigation. Thi Bich Duyen Luu: experimental synthesis; formal analysis; investigation. Thi Bang Tam Dao: resources; data curation. Trung Do Nguyen: resources; instrumentation. Chi Nhan Ha Thuc: resources; investigation; supervision; validation. Hieu Le Van: investigation; supervision; validation; writing – review & editing.

## Conflicts of interest

There are no conflicts to declare.

## Supplementary Material

RA-OLF-D6RA09284E-s001

## Data Availability

The data supporting this article have been included as part of the supplementary information (SI). Supplementary information: experimental setups and data of electrochemical impedance spectroscopy, nitrogen gas adsorption isotherms, tables of tensile properties, and stress–strain curves of tensile measurements. See DOI: https://doi.org/10.1039/d6ra09284e.

## References

[cit1] Li S., Wang X., Guo Y., Hu J., Lin S., Tu Y., Chen L., Ni Y., Huang L. (2022). Recent advances on cellulose-based nanofiltration membranes and their applications in drinking water purification: A review. J. Cleaner Prod..

[cit2] Azimi B., Sepahvand S., Ismaeilimoghadam S., Kargarzadeh H., Ashori A., Jonoobi M., Danti S. (2024). Application of Cellulose-Based Materials as Water Purification Filters; A State-of-the-Art Review. J. Polym. Environ..

[cit3] Islam M. D., Uddin F. J., Rashid T. U., Shahruzzaman M. (2023). Cellulose acetate-based membrane for wastewater treatment—A state-of-the-art review. Mater. Adv..

[cit4] Ahmad N. N. R., Ang W. L., Teow Y. H. (2022). *et al.*, Nanofiltration membrane processes for water recycling, reuse and product recovery within various industries: A review. J. Water Process Eng..

[cit5] Pang L., Meier-Haack J., Huang S., Qi L., Cui H., Ruan S., Zeng Y. J. (2021). Antibiofouling Thin-Film Nanocomposite Membranes for Sustainable Water Purification. Adv. Sustainable Syst..

[cit6] Zhang M., Sun J., Mao Y., Liu G., Jin W. (2019). Effect of substrate on formation and nanofiltration performance of graphene oxide membranes. J. Membr. Sci..

[cit7] Yang L., Xiao X., Shen S., Lama J., Hu M. (2022). *et al.*, Recent advances in graphene oxide membranes for nanofiltration. ACS Appl. Nano Mater..

[cit8] Yang J., Li Z., Wang Z., Yuan S., Li Y., Zhao W., Zhang X. (2021). 2D Material Based Thin-Film Nanocomposite Membranes for Water Treatment. Adv. Mater. Technol..

[cit9] Bhol P., Yadav S., Altaee A., Saxena M., Misra P. K., Samal A. K. (2021). Graphene-Based Membranes for Water and Wastewater Treatment: A Review. ACS Appl. Nano Mater..

[cit10] Zhu L. S., Guo X. X., Chen Y. Q., Chen Z., Lan Y. H., Hong Y. B., Lan W. G. (2022). Graphene Oxide Composite Membranes for Water Purification. ACS Appl. Nano Mater..

[cit11] Lin L., Peng H., Liu Z. (2019). Synthesis challenges for graphene industry. Nat. Mater..

[cit12] Le H. N., Ha Thuc C. N., Le Van H. (2025). Advancements in chemical oxidation-reduction reactions and supramolecular hydrogels of graphene-based materials. Nanotechnology.

[cit13] Le H. N., Luu T. B. D., Pham L. N., Dao T. B. T., Nguyen T. D., Ha Thuc C. N., Le V. H. (2025). Scalable Syntheses of Graphene Oxide and Reduced Graphene Oxide using Cascade Design Oxidation and Highly Basic Reduction Reactions. J. Visualized Exp..

[cit14] Le H. N., Dao T. B. T., Nguyen T. D., Dinh D. A., Ha Thuc C. N., Le V. H. (2024). Revisiting oxidation and reduction reactions for synthesizing a three-dimensional hydrogel of reduced graphene oxide. RSC Adv..

[cit15] Le H. N., Thai D., Nguyen T. T., Dao T. B. T., Nguyen T. D., Tieu D. T., Ha Thuc C. N. (2023). Improving Safety and Efficiency
in Graphene Oxide Production Technology. J. Mater. Res. Technol..

[cit16] Ashok Kumar S. S., Kandiah K. K., Loh K. H., Liew J., Goh Z. L., Ramesh K., Ramesh S., Tiong S. K. (2025). Review: the revolutionary solutions of graphene-based nanomaterials for desalination and water purification membranes and its applications. J. Mater. Sci..

[cit17] Wang X., Mao Y. F., Shen X., Zhao J., Zhou J., Liu Z. (2024). Advances and prospects in graphene oxide membranes for seawater salt ion sieving and rejection. Sep. Purif. Technol..

[cit18] Tiwary S. K., Singh M., Chavan S. V., Karim A. (2024). Graphene oxide-based membranes for water desalination and purification. npj 2D Mater. Appl..

[cit19] Luo W., Chen J., Sun Y., He Z., Li Y., Shi G. (2023). Ultrafast Molecular-Sieving Graphene Oxide Membranes for Selective Separation of Volatile Aromatic Compounds. ACS Appl. Nano Mater..

[cit20] Tiwary S. K., Singh M., Likhi F. H., Dabade S., Douglas J. F., Karim A. (2024). Self-Cross-Linking of MXene-Intercalated Graphene Oxide Membranes with Antiswelling Properties for Dye and Salt Rejection. ACS Environ. Au.

[cit21] Qu H., Xiao X., Han Z., Hu M., Shen S. (2022). *et al.*, Graphene oxide nanofiltration membrane based on three-dimensional size-controllable metal–organic frameworks for water treatment. ACS Appl. Nano Mater..

[cit22] Ye J., Zhang B., Gu Y., Yu M., Wang D., Wu J., Li J. (2019). Tailored Graphene Oxide Membranes for the Separation of Ions and Molecules. ACS Appl. Nano Mater..

[cit23] Mir I. S., Riaz A., Fréchette J. (2024). *et al.*, Bacterial cellulose-graphene oxide composite membranes with enhanced fouling resistance for bio-effluents management. npj Clean Water.

[cit24] Devadiga M. M., Bhat A. S., Sarangi S., HS A. K., Wang N., Tiwari S. K. (2026). Resource-efficient pretreatment with partial desalination approach using graphene-MXene coated cellulose filters for desalination plants. Nanoscale Adv..

[cit25] Sitko R., Musielak M., Zawisza B., Talik E., Gagor A. (2016). Graphene oxide/cellulose membranes in adsorption of divalent metal ions. RSC Adv..

[cit26] Gao W., Wu G., Janicke M. T., Cullen D. A., Mukundan R., Baldwin J. K., Brosha E. L., Galande C., Ajayan P. M., More K. L., Dattelbaum A. M., Zelenay P. (2014). Ozonated Graphene Oxide Film as a Proton-Exchange Membrane. Angew. Chem..

[cit27] Zhang L., Liu Z., Yang C., García Sakai V., Tyagi M., Hong L. (2022). Conduction mechanism in graphene oxide membranes with varied water content: from proton hopping dominant to ion diffusion dominant. ACS Nano.

[cit28] Xu P., Mujumdar A. S., Yu B. (2009). Drying-induced cracks in thin film fabricated from colloidal dispersions. Drying Technol..

[cit29] Le H. N., Nguyen D. K., Dang M. T., Nguyen H. T., Dao T. B. T., Nguyen T. D., Ha Thuc C. N., Le V. H. (2025). Supramolecular hydration structure of graphene-based hydrogels: Density functional theory, green chemistry and interface application. Beilstein J. Nanotechnol..

[cit30] Le H. N., Nguyen T. B. Y., Nguyen D. T. T., Dao T. B. T., Nguyen T. D., Ha Thuc C. N. (2024). Sonochemical synthesis of bioinspired graphene oxide – zinc oxide hydrogel for antibacterial painting on biodegradable polylactide film. Nanotechnology.

[cit31] Le H. N., Nguyen H. T., Dao T. B. T., Nguyen T. D., Tran T. T. V., Ha-Thuc C. N., Le-Van H. (2026). Biomimetic water-mediated nanosilica-zinc hydroxide-polyvinyl alcohol hydrogels for antimicrobial coatings on polylactide and cellulose substrates. Nanotechnology.

[cit32] Le N. H., Seema H., Kemp K. C., Ahmed N., Tiwari J. N., Park S., Kim K. S. (2013). Solution-Processable Conductive Micro-Hydrogels of Nanoparticle/Graphene Platelets Produced by Reversible Self-Assembly and Aqueous Exfoliation. J. Mater. Chem. A.

[cit33] Kuwertz R., Kirstein C., Turek T., Kunz U. (2016). Influence of acid pretreatment on ionic conductivity of Nafion membranes. J. Membr. Sci..

[cit34] Hu R., He Y., Huang M., Zhao G., Zhu H. (2018). Strong Adhesion of Graphene Oxide Coating on Polymer Separation Membranes. Langmuir.

[cit35] Huang H., Park H., Huang J. (2022). Self-crosslinking of graphene oxide sheets by dehydration. Chem.

[cit36] Li W., Wu W., Li Z. (2018). Controlling Interlayer Spacing of Graphene Oxide Membranes by External Pressure Regulation. ACS Nano.

[cit37] Xia S., Ni M., Zhu T., Zhao Y., Li N. (2015). Ultrathin graphene oxide nanosheet membranes with various d-spacing assembled using the pressure-assisted filtration method for removing natural organic matter. Desalination.

[cit38] Thebo K. H., Qian X., Zhang Q., Chen L., Cheng H. M., Ren W. (2018). Highly stable graphene-oxide-based membranes with superior permeability. Nat. Commun..

[cit39] Li A., Li C., Zheng Z., Zhu L., Wang Z., Xie D. (2026). A cellulose nanofiber assembled nanoporous membrane with exceptional dye desalination. Desalination.

[cit40] Lin J., Fu C., Zeng W., Wang D., Huang F., Lin S., Cao S., Chen L., Ni Y., Huang L. (2023). Regulating the structure of cellulose-based ultrafiltration membrane to improve its performance for water purification. Ind. Crops Prod..

[cit41] Yan L., Chen J., Zhang Z., Liu Z., Ding T., Shi G. (2025). Reduced graphene oxide membrane with small nanosheets for efficient and ultrafast removal of both microplastics and small molecules. J. Hazard. Mater..

[cit42] Guan J., You X., Shi B., Liu Y., Yuan J., Yang C., Pang X., Wu H., Shen J., Fan C., Long M., Zhang R., Jiang Z. (2021). Engineering multi-pathway graphene oxide membranes toward ultrafast water purification. J. Membr. Sci..

[cit43] Ma X., Wang A., Miao J., Fan T. (2023). 2D lamellar membrane with MXene hetero- intercalated small sized graphene oxide for harsh environmental wastewater treatment. Sep. Purif. Technol..

[cit44] Zhang W., Xu H., Xie F., Ma X., Niu B., Chen M., Zhang H., Zhang Y., Long D. (2022). General synthesis of ultrafine metal oxide/reduced graphene oxide nanocomposites for ultrahigh-flux nanofiltration membrane. Nat. Commun..

[cit45] Du Y. C., Huang L. J., Wang Y. X., Yang K., Zhang Z. J., Wang Y., Kipper M. J., Belfiore L. A., Tang J. G. (2020). Preparation of graphene oxide/silica hybrid composite membranes and performance studies in water treatment. J. Mater. Sci..

[cit46] Zhu B., Li N., Guo C., Liu P., Li T., Wang L., Xu Z. (2024). LEGO brick-inspired ultra-stable and rapid transport 2D membrane for fast water purification. J. Mater. Chem. A.

[cit47] Vu N. A., Le P. N. P., Nguyen V. N., Tran T. T. V., Ha Thuc C. N., Le V. H. (2021). Effect of imidazole-doped nanocrystalline cellulose on the characterization of Nafion films of fuel cells. J. Chem. Technol. Biotechnol..

[cit48] Su P., Wang F., Li Z., Tang C. Y., Li W. (2020). Graphene oxide membranes: controlling their transport pathways. J. Mater. Chem. A.

[cit49] Feng M., Li N., Zhang Y., Cui Y., Chen Y., Zhang Z., Zhuo Y., Du H., Zhang Y. (2026). Two-dimensional nanofluidics in graphene oxide laminar membranes bridging multiscale channel design and nanoconfined transport. Sep. Purif. Technol..

[cit50] Koseki K., Arita T., Tabata K., Nohara T., Sato R., Nagano S., Masuhara A. (2021). Effect of Surface Silanol Density on the Proton Conductivity of Polymer-Surface-Functionalized Silica Nanoparticles. ACS Sustain. Chem. Eng..

[cit51] Kumar A., Ramani V. (2011). Proton Conducting Silica Particles: Relation between Morphology and Conductivity. ECS Trans..

[cit52] Karim M. R., Hatakeyama K., Matsui T., Takehira H., Taniguchi T., Koinuma M., Matsumoto Y., Akutagawa T., Nakamura T., Noro S. I., Yamada T., Kitagawa H., Hayami S. (2013). Graphene oxide nanosheet with high proton conductivity. J. Am. Chem. Soc..

