# Supplementary material for: Multilayer membranes of graphene oxide nanosheets on cellulose filter papers for high-flux water filtration and high proton conductivity
Source: RSC Adv. 2026 Oct 19;16(48):64227–43. doi: 10.1039/d6ra09284e (PMC13678592; doi:10.1039/d6ra09284e)
Supplement: Supplementary file 1 [file RA-016-D6RA09284E-s001.pdf]

Supplementary Information (SI) for RSC Advances.
This journal is © The Royal Society of Chemistry

Supporting Information

Multilayer membranes of graphene oxide nanosheets on cellulose filter papers for high-flux water filtration and high proton conductivity

Hon Nhien Le[ab*], Ngoc Quang Tran[bde], Nang An Vu[abc], Thi Bich Duyen Luu[ab], Lam Nhu Pham[ab], Thi Bang Tam Dao[ab], Trung Do Nguyen[ab], Chi Nhan Ha Thuc[ab*], and Hieu Le Van[abc*]

[a.] Faculty of Materials Science and Technology, University of Science, Ho Chi Minh City, 700000, Vietnam.

[b.] Vietnam National University, Ho Chi Minh City, 700000, Vietnam.

[c.] Multifunctional Materials Laboratory, University of Science, Ho Chi Minh City, 700000, Vietnam.

[d.] Center for Innovative Materials and Architectures, Ho Chi Minh City, 700000, Vietnam

[e.] Center for Materials Innovation and Technology and College of Engineering and Computer Science, VinUniversity, Gia Lam district, Hanoi, 10000, Vietnam

* Corresponding author

Email address: lhnhien@hcmus.edu.vn (Hon Nhien Le); htcnhan@hcmus.edu.vn (Chi-Nhan Ha-Thuc); lvhieu@hcmus.edu.vn (Hieu Le-Van)

(A) (B)

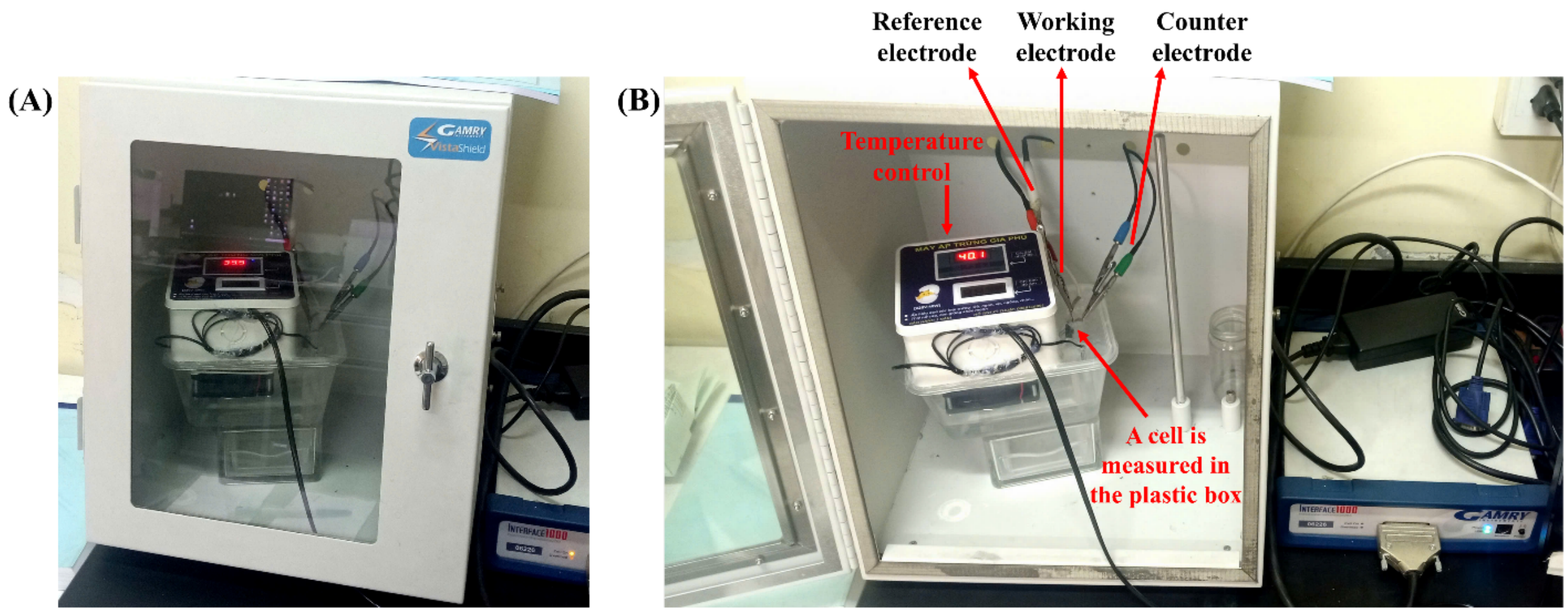

Figure S1. (A) The setup of EIS measurements at 100%RH and various temperatures. (B) The cell assembly of a proton-conducting membrane and two nickel electrodes was put in a plastic box equipped with a temperature controller for EIS measurements at 100%RH and 40°C.

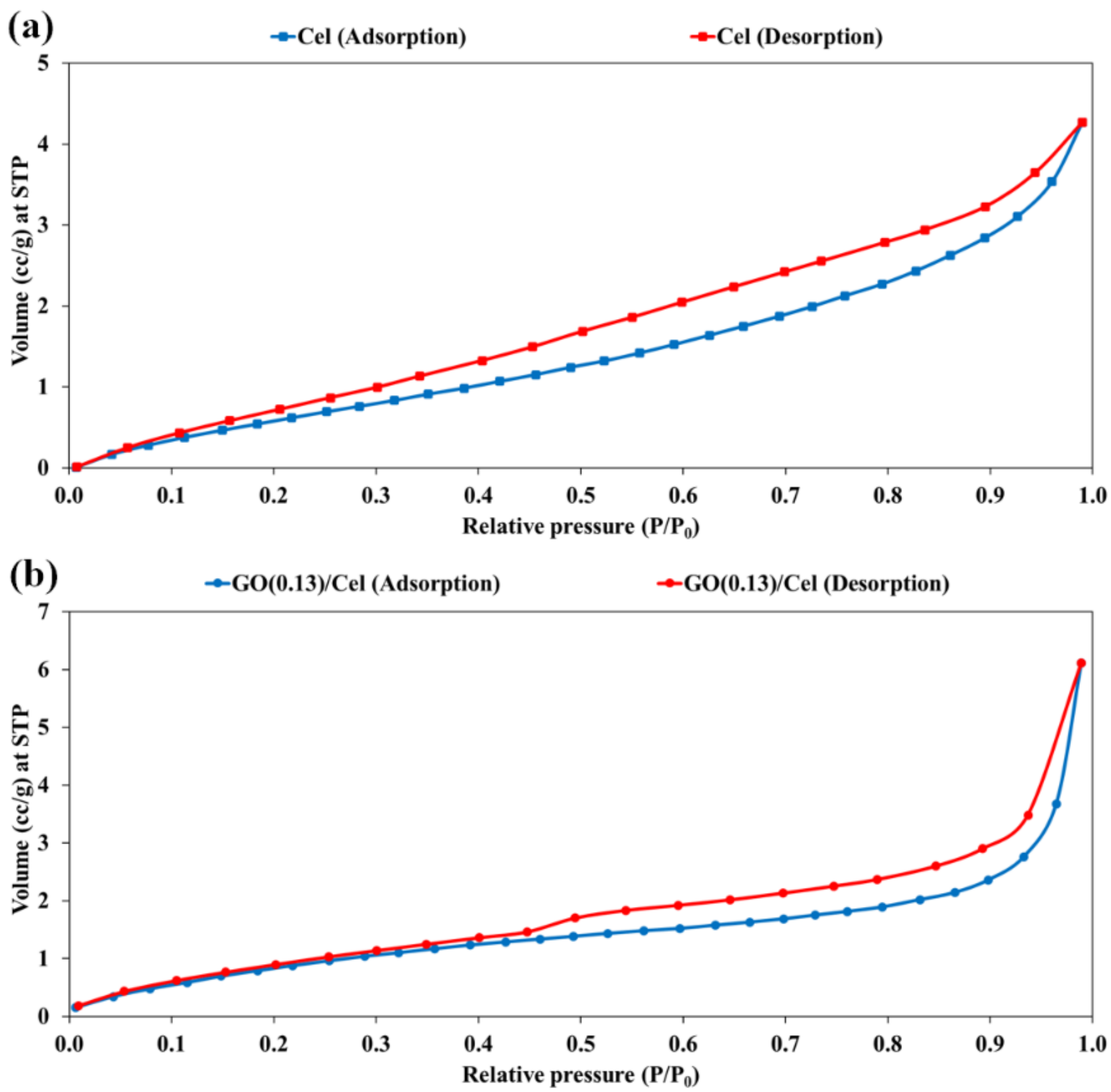

Figure S2. Nitrogen gas adsorption analysis at 77 K. (a) Adsorption and desorption isotherms of Cel (the Cel isotherms are derived from our previous studies [1,2]). (b) Adsorption and desorption isotherms of GO(0.13)/Cel.

Table S1. Tensile properties of GO/cellulose and sandwich membranes. The values of Cel membrane are obtained from our previous paper [2].

Membranes	Tensile strength (MPa)	Elastic modulus (MPa)	Tensile elongation (%)	Energy to break (kgf·mm)
Cel	8.55 ± 0.58	679.14 ± 80.73	6.34 ± 0.42	0.77 ± 0.09
GO(0.07)/Cel	8.63 ± 0.39	1081.96 ± 126.8	4.14 ± 0.36	0.56 ± 0.08
GO(0.13)/Cel	10.35 ± 0.88	1348.24 ± 113.13	5.78 ± 0.66	0.94 ± 0.16
Cel/GO(0.07)/Cel	8.15 ± 1.11	707.78 ± 89.09	6.95 ± 1.29	1.86 ± 0.58
Cel/GO(0.13)/Cel	9.36 ± 0.25	719.37 ± 42.6	6.89 ± 0.32	2.01 ± 0.08

No.	Maximum Load N	Elasticity Modulus MPa	Max Load Stress MPa	Max Load Strain strain	Max Load Elong %	Break Elong %	Break Energy kgf-mm
1	9.15	1220.04	8.97	0.03	2.93	3.99	0.56
2	8.60	926.79	8.43	0.03	3.04	4.17	0.56
3	8.50	981.71	8.33	0.03	2.71	3.98	0.51
4	8.45	1186.64	8.28	0.03	2.88	3.80	0.50
5	9.30	1094.62	9.12	0.04	3.54	4.74	0.69
Average	8.80	1081.96	8.63	0.03	3.02	4.14	0.56
Std	0.40	126.80	0.39	0.00	0.31	0.36	0.08

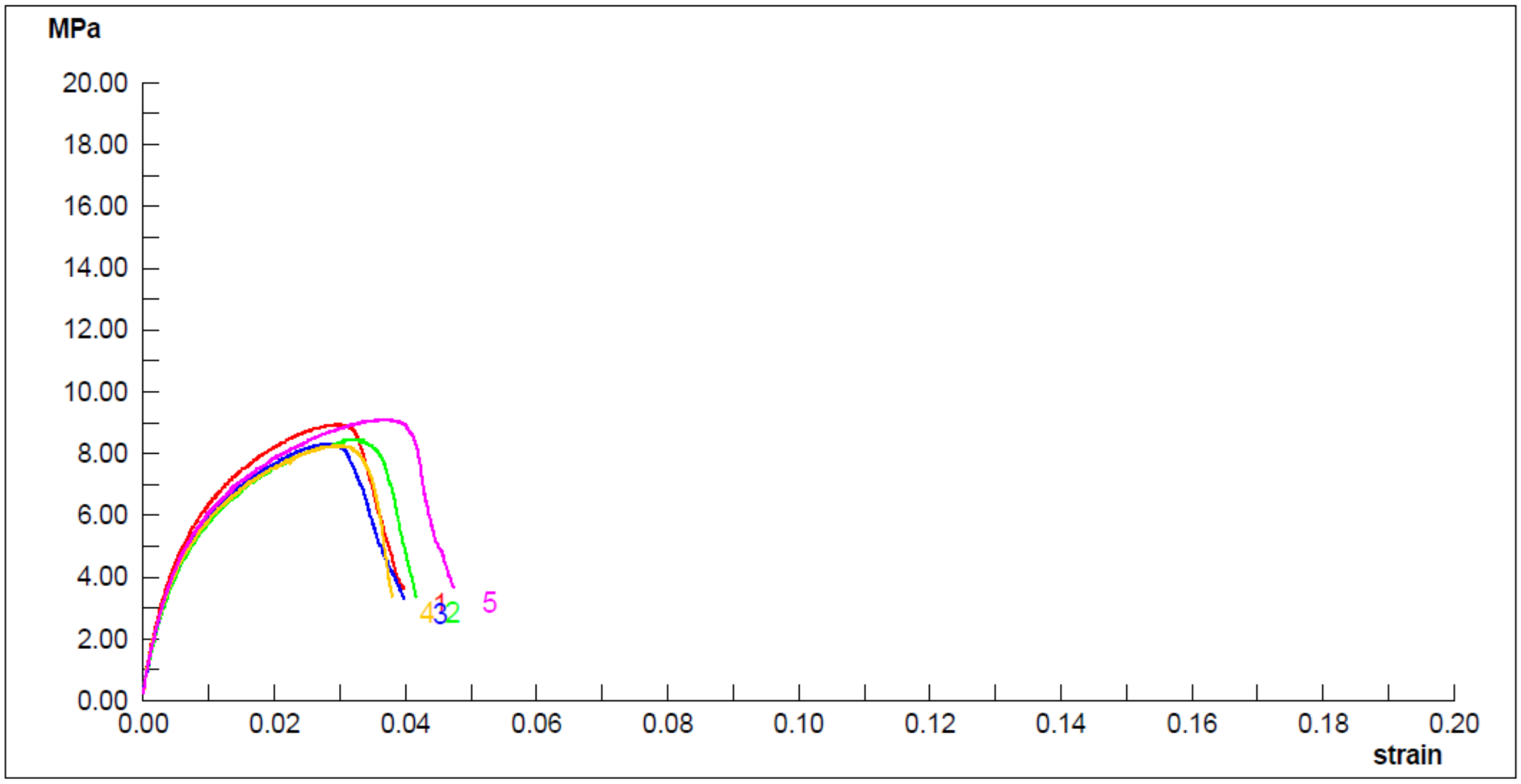

Figure S3. Tensile testing data and stress – strain curves of GO(0.07)/Cel membranes.

No.	Maximum Load N	Elasticity Modulus MPa	Max Load Stress MPa	Max Load Strain strain	Max Load Elong %	Break Elong %	Break Energy kgf-mm
1	11.00	1506.95	10.78	0.04	4.16	5.36	0.93
2	11.00	1382.42	10.78	0.04	4.28	5.97	1.01
3	10.75	1374.71	11.20	0.04	4.33	5.45	0.94
4	10.25	1232.42	10.05	0.04	4.40	6.85	1.12
5	8.60	1244.70	8.96	0.04	3.82	5.28	0.69
Average	10.32	1348.24	10.35	0.04	4.20	5.78	0.94
Std	1.01	113.13	0.88	0.00	0.23	0.66	0.16

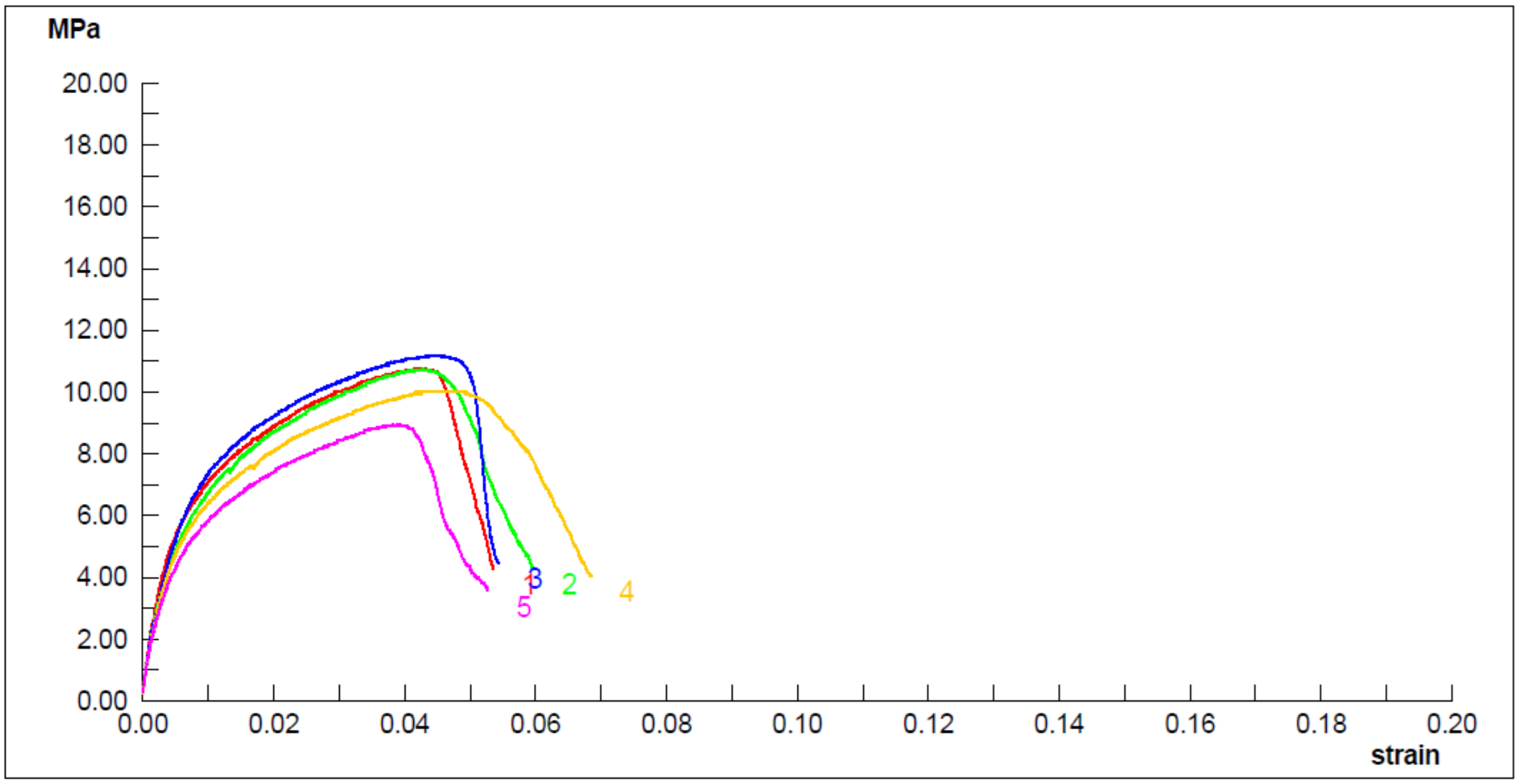

Figure S4. Tensile testing data and stress – strain curves of GO(0.13)/Cel membranes.

No.	Maximum Load N	Elasticity Modulus MPa	Max Load Stress MPa	Max Load Strain strain	Max Load Elong %	Break Elong %	Break Energy kgf-mm	Sample Thickness mm
1	14.20	613.24	6.88	0.04	3.73	5.56	1.20	0.350
2	18.80	790.17	8.95	0.05	5.29	7.16	2.06	0.350
3	18.10	719.93	8.62	0.06	5.69	8.12	2.31	0.350
Average	17.03	707.78	8.15	0.05	4.90	6.95	1.86	0.350
Std	2.48	89.09	1.11	0.01	1.04	1.29	0.58	0.000

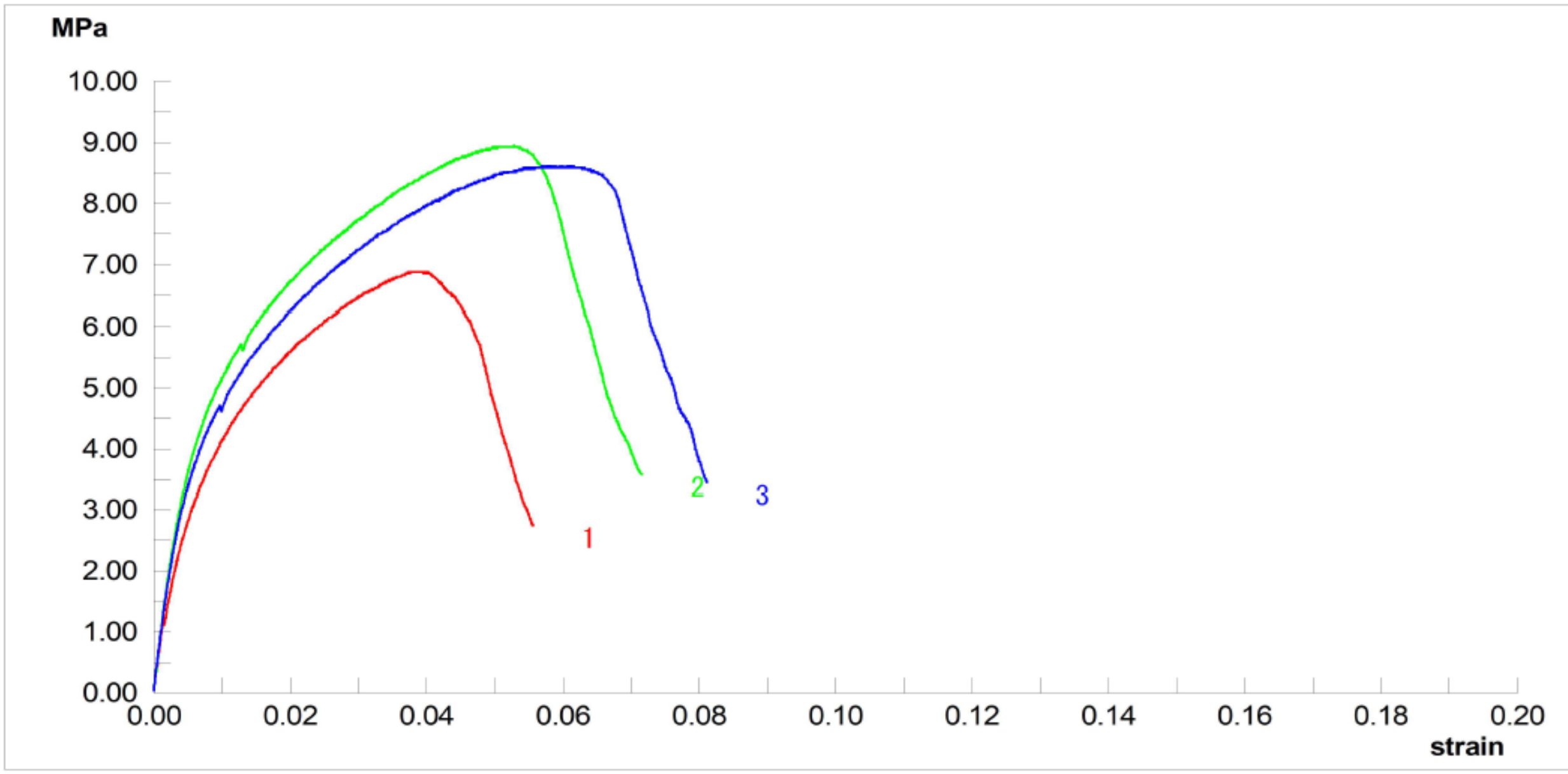

Figure S5. Tensile testing data and stress – strain curves of Cel/GO(0.07)/Cel membranes.

No.	Maximum Load N	Elasticity Modulus MPa	Max Load Stress MPa	Max Load Strain strain	Max Load Elong %	Break Elong %	Break Energy kgf-mm
1	19.05	768.01	9.07	0.04	4.03	7.14	2.04
2	19.55	701.43	9.47	0.05	4.67	6.53	1.92
3	19.70	688.68	9.54	0.05	4.89	7.01	2.07
Average	19.43	719.37	9.36	0.05	4.53	6.89	2.01
Std	0.34	42.60	0.25	0.01	0.45	0.32	0.08

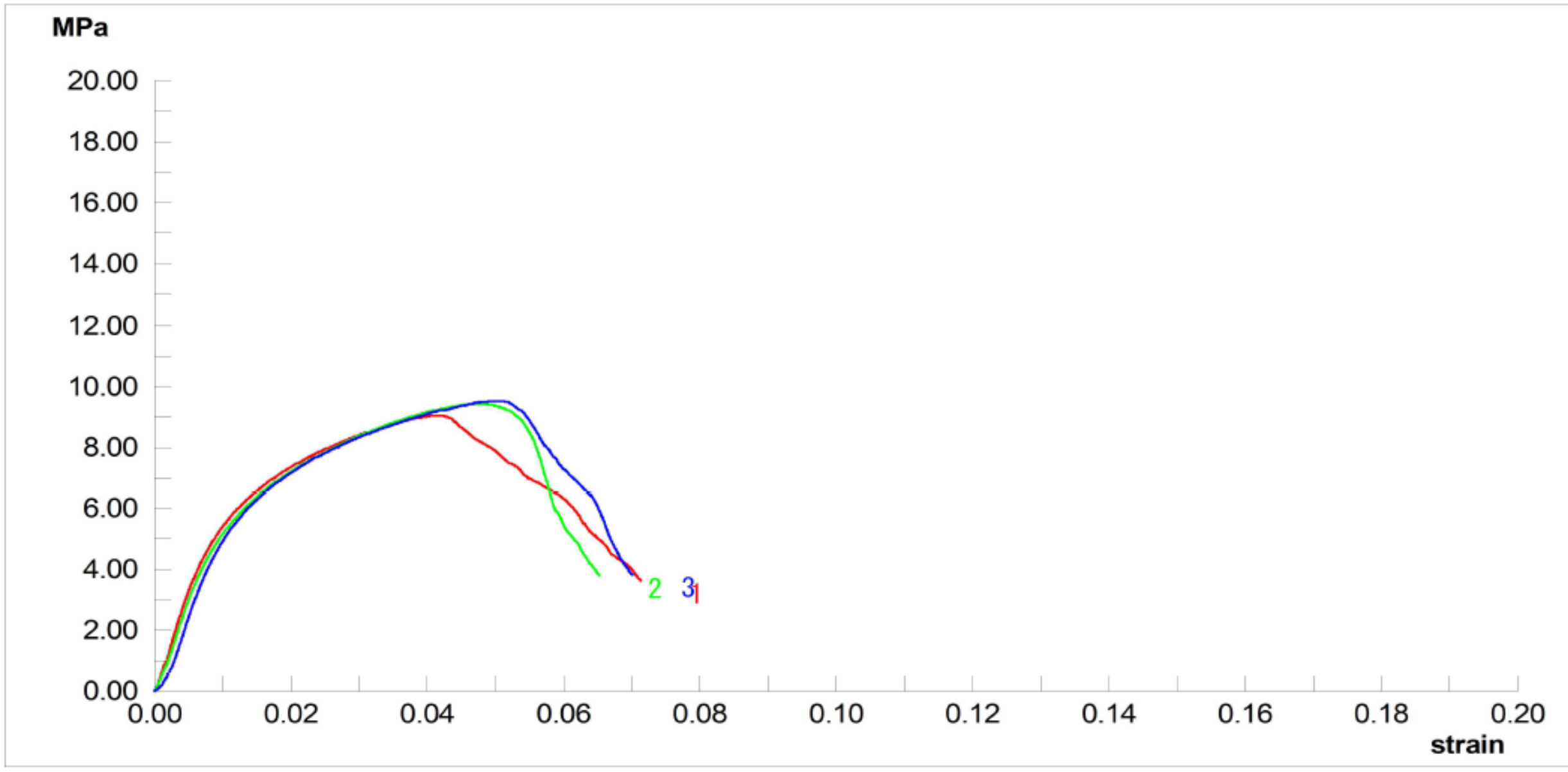

Figure S6. Tensile testing data and stress – strain curves of Cel/GO(0.13)/Cel membranes.

Table S2. Equivalent circuit components and goodness of fit derived from the fitting of EIS data using Gamry Echem Analyst softwate.

Parameters / Membranes	R1 (Ω)	R3 (Ω)	L4 (H)	R5 (□)	Y07	a8	Y09	a10	Goodness of Fit

Cel/Cel at 25ºC	3396	70.12	0	152300	4.438 × 10^{-6}	4.438 × 10^{-6}	4.438 × 10^{-6}	4.438 × 10^{-6}	0.002041
Cel/Cel at 30ºC	56210	57.03	1.556 × 10^{-9}	1481	1.325 × 10^{-6}	793.8 × 10^{-3}	43.60 × 10^{-6}	154.6 × 10^{-3}	0.002053
Cel/Cel at 35ºC	3521	37.18	25.85 × 10^{-15}	192300	189.1 × 10^{-9}	427.1 × 10^{-3}	1.189 × 10^{-6}	792.3 × 10^{-3}	0.001839
Cel/Cel at 40ºC	1702	30.98	0	486100	1.019 × 10^{-9}	826.0 × 10^{-3}	14.43 × 10^{-6}	663.0 × 10^{-3}	0.0002309
Cel/Cel at 45ºC	4428	15.66	430.5 × 10^{-12}	262500	1.728 × 10^{-9}	723.2 × 10^{-3}	1.181 × 10^{-6}	779.8 × 10^{-3}	0.001544
Cel/GO(0.07)/Cel at 25ºC	658.2	8.008	3.974 × 10^{-9}	8400	47.95 × 10^{-9}	606.2 × 10^{-3}	27.63 × 10^{-6}	756.4 × 10^{-3}	0.0007398
Cel/GO(0.07)/Cel at 30ºC	687.2	6.55	63.47 × 10^{-18}	17380	113.5 × 10^{-9}	552.3 × 10^{-3}	28.62 × 10^{-6}	764.7 × 10^{-3}	0.0007139
Cel/GO(0.07)/Cel at 35ºC	445.1	5.181	1.290 × 10^{-9}	3377	886.5 × 10^{-9}	417.1 × 10^{-3}	16.45 × 10^{-6}	827.1 × 10^{-3}	0.0005629
Cel/GO(0.07)/Cel at 40ºC	55180	4.159	1.279 × 10^{-12}	3001	1.277 × 10^{-6}	803.1 × 10^{-3}	236.8 × 10^{-6}	73.76 × 10^{-3}	0.002143
Cel/GO(0.07)/Cel at 45ºC	33.90 × 10^{9}	2.674	11.91 × 10^{-9}	47380	1.124 × 10^{-3}	29.50 × 10^{-3}	24.67 × 10^{-6}	799.0 × 10^{-3}	0.002969
Cel/GO(0.13)/Cel at 25ºC	365.7	4.408	29.84 × 10^{-18}	2021	3.646 × 10^{-6}	330.0 × 10^{-3}	44.54 × 10^{-6}	832.6 × 10^{-3}	0.000413

Cel/GO(0.13)/Cel at 30°C	501.1	3.507	3.778×10^{-12}	20970	912.4×10^{-9}	422.6×10^{-3}	53.04×10^{-6}	790.2×10^{-3}	0.0008751
Cel/GO(0.13)/Cel at 35°C	857.0	2.494	0	38720	30.74×10^{-9}	599.9×10^{-3}	19.65×10^{-6}	730.0×10^{-3}	0.000400
Cel/GO(0.13)/Cel at 40°C	511.2	1.957	1.181×10^{-6}	24230	49.44×10^{-9}	613.2×10^{-3}	54.02×10^{-6}	780.6×10^{-3}	0.0008094
Cel/GO(0.13)/Cel at 45°C	14380	1.73	827.2×10^{-18}	9907	32.86×10^{-6}	711.2×10^{-3}	751.1×10^{-6}	30.39×10^{-3}	0.003004

Table S3. EIS measurement parameters, proton conductivities, and activation energies of the sandwich membranes.

Proton-conducting membrane	Membrane thickness (mm)	Electrode area (mm^2)	R_{mem} (Ω)	Temperature (°C)	Proton conductivty (mS cm^{-1})	Activation energy (KJ mol^{-1})
Cel/Cel	0.37	49	70.12	25	1.08	56.67
	0.37	49	57.03	30	1.32	
	0.37	49	37.18	35	2.03	
	0.37	49	30.98	40	2.44	
	0.37	49	15.66	45	4.82	
Cel/GO(0.07)/Cel	0.375	49	8.008	25	9.56	41.60
	0.375	49	6.55	30	11.68	
	0.375	49	5.181	35	14.77	
	0.375	49	4.159	40	18.40	
	0.375	49	2.674	45	28.62	
	0.38	49	4.408	25	17.59	38.74

Cel/GO(0.13)/Cel	0.38	49	3.507	30	22.11	
	0.38	49	2.494	35	31.10	
	0.38	49	1.957	40	39.63	
	0.38	49	1.73	45	44.83	

Reference

[1] H.N. Le, L.N. Pham, T.B.D. Luu, T.B.T. Dao, T.D. Nguyen, C.N. Ha Thuc, V.H. Le, Conversion of rice husk ash into water-lubricated nanosilica hydrogel for brush coating on cellulose paper for water filtration, VNUHCM Journal of Science and Technology Development, 2026, 29, 4051-4064.

[2] H.N. Le, H.T. Nguyen, T.B.T. Dao, T.D. Nguyen, T.T.V. Tran, C.N. Ha-Thuc, H. Le-Van, Biomimetic water-mediated nanosilica-zinc hydroxide-polyvinyl alcohol hydrogels for antimicrobial coatings on polylactide and cellulose substrates, Nanotechnology, 2026, 37, 235602. https://doi.org/10.1088/1361-6528/ae6dcb
